# From Traditional Medicine to the Laboratory: A Multidisciplinary Investigation on *Agrimonia eupatoria* L. Collected in Valle Imagna (BG, North of Italy)

**DOI:** 10.3390/plants14030340

**Published:** 2025-01-23

**Authors:** Fabrizia Milani, Chiara Muratore, Sara Biella, Martina Bottoni, Elio Rossi, Lorenzo Colombo, Paola Sira Colombo, Piero Bruschi, Alessio Papini, Paolo Landini, Claudia Giuliani, Fabrizio Araniti, Bhakti Prinsi, Gelsomina Fico

**Affiliations:** 1 Department of Pharmaceutical Sciences, Università degli Studi di Milano, Via Luigi Mangiagalli 25, 20133 Milan, Italy; fabrizia.milani@unimi.it (F.M.); lorecolo.93@gmail.com (L.C.); pasico19@virgilio.it (P.S.C.); claudia.giuliani@unimi.it (C.G.); gelsomina.fico@unimi.it (G.F.); 2 “G.E. Ghirardi” Botanical Garden, Department of Pharmaceutical Sciences, Università degli Studi di Milano, Via Religione 25, 25088 Toscolano Maderno, Italy; 3 Department of Agricultural and Environmental Sciences—Production, Landscape, Agroenergy, Università degli Studi di Milano, Via Celoria 2, 20133 Milan, Italy; chiara.muratore@unimi.it (C.M.); fabrizio.araniti@unimi.it (F.A.); bhakti.prinsi@unimi.it (B.P.); 4 Department of Biosciences, Università degli Studi di Milano, Via Celoria 26, 20133 Milan, Italy; sara.biella@unimi.it (S.B.); elio.rossi@unimi.it (E.R.); paolo.landini@unimi.it (P.L.); 5 Department of Agricultural, Environmental, Food and Forestry Science and Technology, Università degli Studi di Firenze, Piazzale delle Cascine 18, 50144 Florence, Italy; piero.bruschi@unifi.it; 6 Department of Biology, Università degli Studi di Firenze, Via La Pira 4, 50121 Florence, Italy; alessio.papini@unifi.it

**Keywords:** *Agrimonia eupatoria* L. subsp. *eupatoria*, Valle Imagna, wound healing, glandular trichomes, phenolic compounds, volatile organic compounds, antimicrobial activity, adhesion modulation

## Abstract

A previous ethnobotanical investigation conducted in Valle Imagna (Northern Italy) highlighted the traditional use of *Agrimonia eupatoria* L. (Rosaceae) as a disinfectant and wound-healing agent. This use seemed to be linked to a local 18th century manuscript. This species was chosen for a multidisciplinary investigation to validate or refute its traditional use in the valley. Samples from fresh leaves were observed under Scanning Electron and Light Microscopy. The phenolic profiles of an epicuticular aqueous extract of the whole leaves and of infusions and decoctions of leaves and aerial parts were analyzed through Liquid Chromatography–Tandem Mass Spectrometry. The volatile organic compounds (VOCs) of fresh leaves were analyzed through Headspace Solid-Phase Microextraction coupled with Gas Chromatography–Mass Spectrometry. Growth inhibition and adhesion modulation were assessed on *Escherichia coli*, *Staphylococcus aureus*, and *S. warneri* by minimum inhibitory concentration and adhesion assays. Two trichome morphotypes were observed: a capitate with a one-celled rounded head and a capitate with a teo-celled cylindrical head. Both were responsible for producing terpenes, while the cylindrical capitates also produced polyphenols. Thirty-four phenolic compounds were characterized. Luteolin-7-O-glucoside, Catechin, and Epicatechin were common to all five extracts. The VOC profiles highlighted the dominance of (+)-α-Pinene. The infusions and the decoctions had a significant inhibitory activity on *E. coli*, and the extracts (specifically, the infusion of the leaves and both decoctions) also had a stimulating effect on the biofilm formation of *S. warneri*. These results already hold particular interest because of the strong connection they have to the traditional use of agrimony described in Valle Imagna.

## 1. Introduction

*Agrimonia eupatoria* L. (Rosaceae) is a perennial herbaceous species common to the temperate regions of the world. It grows especially on sunny meadows and fallows, usually from sea levels up to 1000 m a.s.l. It presents a short rhizome and erect and pubescent stems, which can be woody at the base. The leaves, odd-pinnate with a toothed margin, are usually collected in a basal rosette but also grow on the stems during the second year. The yellow hermaphrodite flowers are gathered along spikes and have five yellow petals and five sepals, five stamens and a superior ovary. The fruits are achenes included in a *hypantium* covered in bristles [1,2,3]. In Europe, three subspecies are recognized based on macroscopic diagnostic features (such as the ratio between *hypantium* and the crown’s length or the angle taken by the external bristles in relation to the *hypantium*’s longitudinal axis): *A. eupatoria* subsp. *eupatoria* L., *A. eupatoria* subsp. *grandis* (Andrz. Ex Asch. & Graebn.), and *A. eupatoria* subsp. *asiatica* (Juz.) Skaliký [1,2,3]. According to Iamonico, 2011, the nominal subspecies is the most common in Italy; the occurrence of *A. eupatoria* subsp. *grandis* is confirmed only in Calabria as its presence is considered historical in Lombardy, Basilicata, and Sicily and even dubious in Piedmont and Veneto. The presence of *A. eupatoria* subsp. *asiatica* has never been reported in Italy [2].

In the literature, the species was described in Europe (Bulgaria, Czech Republic, Germany, Poland, Serbia, and Turkey) as being traditionally used as an infusion, often of the aerial parts, acting as astringent and anti-inflammatory agents for diarrhea and gingivitis, as well as a wound-healing agent. The infusion was taken orally or through gargling or would be applied as compresses [4]. Additionally, in England, it was considered a valid ingredient for wound-healing remedies, as found in medical textbooks from the 10th century [5]. In Northern Italy, the species was mentioned by Vitalini and their group in 2015 (infusion as compresses for mild dermatitis or drunk as an astringent) and in 2009 (an infusion of the leaves for laryngitis) [6,7].

Furthermore, in the pre-alpine valley of Valle Imagna (the province of Bergamo, the North of Italy), the use of *A. eupatoria* as an anti-inflammatory agent, as well as a helpful wound-healing agent, was recorded both in historical sources [8] and during the field ethnobotanical investigation that we conducted from October 2021 to November 2022 and reported in Milani et al. [9]. The preliminary results obtained during this field investigation prompted us to choose *A. eupatoria* as the first target species of this territory for a subsequent multi-step laboratory analysis guided by both the traditional uses highlighted in the valley and the scientific literature currently dedicated to this species.

From a micromorphological point of view, this species has been little studied so far: in 2017, Faghir et al. investigated the tribe *Agrimoniae* (including the three subspecies of *A. eupatoria* subsp. *grandis*, *A. eupatoria* subsp. *eupatoria*, and *A. eupatoria* subsp. *asiatica*) under Scanning Electron (SEM) and Light Microscopy (LM) to demonstrate that some micromorphological features of both glandular and non-glandular *indumenta* could be used to discriminate the different subspecies [10]. At the moment, no information has been found concerning a histochemical analysis on the species.

As regards phytochemistry, different types of extracts have been analyzed and can be found in the literature, even if they are rarely related to the traditional uses detected in various territories. The aerial parts represent a part of the plant commonly investigated in the literature. The polyphenolic fraction has been characterized in extracts obtained by solvents at various degrees of polarity, such as acetone ([11], in which luteolin-7-O-glucoside, hyperoside, and apigenin glycoside were the largest peaks identified), ethyl acetate (as a fraction of an aqueous extract, in which flavan-3-ols, quercetin, and kaempferol derivatives were especially identified [12,13]), methanol (where the more abundant compounds were rutin, hyperoside, and isoquercitrin [11,14,15,16,17]), and water (with isoquercitrin, agrimoniin, hyperoside, cynaroside, and isoquercitroside among the most abundant compounds [11,15,18,19]). The essential oil profiles from leaves and roots were investigated by Feng and their group in 2013 [20]. To the best of our knowledge, the epicuticular depositions on the surfaces of whole leaves, whether fresh or dried, as well as the volatile organic compounds (VOCs) spontaneously emitted by fresh leaves, have never been investigated.

Finally, from a pharmacological standpoint, various authors have assessed the potential biological activities of *A. eupatoria* and at different apparatus levels: antibacterial and antibiofilm [5,21,22,23,24], anti-fungal and antiviral [24,25], antidiabetic [26], anti-inflammatory and antioxidant [4], anti-tumor and citotoxic [27], hepatoprotective [28], neuroprotective [16], and wound healing [29,30].

From the analysis of the scientific literature, the general impression that we obtained was that there was a gap concerning the species’ micromorphology and histochemistry, and the results, for the most part, were fragmented and fundamentally inconsistent with the traditional uses of the species where phytochemical and pharmacological investigations were concerned.

For these reasons, the work presented herein has the aim of presenting the following: (*a*) the comprehensive and detailed raw primary data obtained from our previous ethnobotanical investigation conducted in Valle Imagna concerning solely the traditional uses of the species *A. eupatoria* in that territory; (*b*) a micromorphological investigation on fresh leaves and, as an element of novelty in the literature, histochemical analyses; (*c*) a phytochemical survey concerning both phenolic and terpenic fractions conducted on extracts obtained by taking into account the traditional preparations of the territory of Valle Imagna: infusions and decoctions of both grounded leaves and grounded aerial parts, aqueous (35 °C) and acetonic epicuticular extract of whole leaves, and the VOCs spontaneously emitted by fresh leaves; and (*d*) a preliminary screening on the antibacterial and adhesion modulatory activities of the traditional water extracts obtained during step (*c*) on *Escherichia coli* MG1655, *Staphylococcus aureus*, and *S. warneri*.

## 2. Results and Discussion

### 2.1. Raw Primary Data on the Species

As we reported in our previous work [9], between October 2021 and November 2022, we conducted an ethnobotanical investigation in the territory of upper Valle Imagna (Bergamo, the North of Italy). The total number of use reports (URs) obtained from the 109 local informants and recorded in the database was 3844, with the food sector coming first in terms of the number of URs with 2296 and the medicinal sector coming second with 670 URs. In the medicinal sector, the most reported category of pathology was ‘Skin diseases and traumas’.

In the framework of this field investigation, *A. eupatoria* (vernacular name ‘*Erba del Venil*’ or ‘*Erba del Vinil*’) was the third most reported species in the medicinal field (the number of URs = 27, after *Malva sylvestris* L. n = 135 and *Tilia cordata* Mill. n = 52) and third in the category ‘Skin diseases and traumas’ (n = 10, after *Hypericum perforatum* L. n = 21, and *M. sylvestris* n = 11). Specifically, in this sector, *A. eupatoria* was mentioned by 10 informants (5 women and 5 men), each of whom reported at least one medicinal use.

In this category, the species was reported as ‘Anti-inflammatory, disinfectant, and wound healing’ (n = 10). In particular, leaves were the most used part of the plant (n = 7), followed by the whole plant (specifically the epigeal parts with the first part of the rhizomes, n = 2), and aerial parts (n = 1). The most cited preparation forms were ‘none, used as it is’ (n = 4), infusion (n = 4), and decoction (n = 2). The way of administration in this category was external, manifesting as compresses (n = 6) or as fresh leaves applied (n = 4).

To provide more detail, leaves were applied directly on the wounds that were considered by the informants ‘*deep, hard to treat otherwise*’ or they were lightly crushed (without breaking them). One of the informants, a local medical doctor, clearly remembered that in one of the municipalities (Costa Valle Imagna), the species was used by the elders solely for the application of the fresh leaf, which was then kept in place with a gauze. The leaf needed to be frequently changed and kept in contact with the wound for at least 3–4 days. The leaves or the whole plant were also used to prepare infusions or decoctions that were then used for external compresses with wound-healing properties, applied directly to wounded or inflamed skin.

In the diachronic analysis of historical sources that we previously conducted in 2023 [9], we highlighted that *A. eupatoria* was 1 of the 12 remedies found in both a XVIII century manuscript found in the valley [8] and the field investigation conducted with the informants [9]. *A. eupatoria* was the main ingredient of a remedy applied to deep leg ulcers. The wounds were disinfected with a wine decoction of aerial parts of *A. eupatoria* and dried roses. The cooked sediments of agrimony plants were then applied to the same wounds and kept in place with a gauze. It was indeed this diachronic comparison that prompted us to choose agrimony as the target species for the study presented herein.

*A. eupatoria* was also one of the species found by Watkins et al. in 10th century Anglo-Saxon medical texts as an ingredient (specifically, fresh or dried aerial parts and roots) of, among others, salves for the treatment of ulcerated sores and wounds [5].

It is interesting to highlight that in other ethnobotanical investigations conducted in Northern Italy, and specifically in Lombardy, this species was rarely cited and, when cited, its traditional uses did not overlap with what we found in Valle Imagna. As a matter of fact, Vitalini et al. reported *A. eupatoria* as useful to treat laryngitis in 2009, while compresses of the infusion were topically applied in cases of mild dermatitis in 2015 [6,7]. It is possible to hypothesize that the knowledge coming from the historical sources found in Valle Imagna specifically blended throughout the centuries into the traditional knowledge of this territory, providing it with a peculiar identity, different from other territories of the Lombardy region.

Considering the other categories of pathology, the other 17 URs were distributed as follows: digestive system problems (n = 5), urinary tract disorders (n = 5), oro-pharyngeal affections (n = 2), general condition (n = 2), early-infancy disorders (n = 1), and musculoskeletal traumas (n = 1).

The use of agrimony was considered ‘past/disappeared’ by seven informants, while it was still considered ‘current’ by three of them. This kind of information was actually common to many other plant species mentioned. In fact, the collection and use of this species appeared to be very common until the 1950s, 60s, and 70s, periods that coincide with decades of great migrations from Valle Imagna to Switzerland, France, and Germany due to the extended poverty that characterized the valley in the aftermath of World War 2. It clearly appeared during the interviews that many of the elderly of Valle Imagna did not have pleasant memories of that period and the rural life they used to conduct, and by leaving the valley during that time, they had left behind not only their lives and territory but also everything else connected to it, including plant species and their traditional uses.

**Figure 1:**
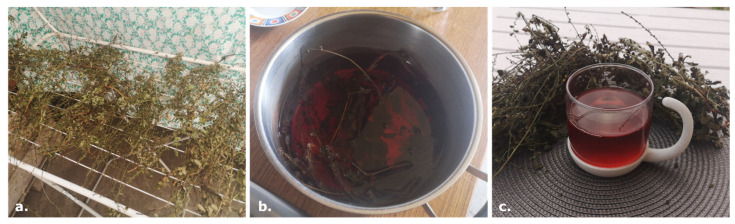
Pictures from an interview with SAN005 and SAN006. Aerial parts of *A. eupatoria* are collected and left to dry on a drying rack in a cool and aerated place (**a**). The hybrid method using infusion and decoction is then conducted in the kitchen (**b**) and the resulting mixture is drunk either warm or cool (**c**).

The two informants that still used the species (SAN005 and SAN006) showed us their method of preparation, which was almost a hybrid between infusion and decoction. They would put the whole epigeal parts with the first part of the roots in cool water (sometimes, they would also add leaves of *M. sylvestris*), bring the water to a boil, and finally remove the pot from the stove immediately after the water started boiling. The preparation was then left to rest for 15–20 min before being filtered and drunk (Figure 1).

Further information concerning every UR recorded for *A. eupatoria* in the medicinal sector is reported here for the first time in Table 1 in a comprehensive and detailed way.

From Table 1, it is clear that the plant and its uses are especially remembered by the elderly of the community. It is important to highlight that some of the other informants remembered a plant called ‘*Erba del Vinil*’, but they could not report its specific use. In fact, some of them were able to remember observing their parents or grandparents collecting the species when they were growing up, but they could not remember how it was prepared and used (i.e., ‘I remember my aunt would use *Erba del Vinil* to cure any problem, sometimes even for the animals’).

To complete the description of the use of this species, *A. eupatoria* was also reported 4 times in the veterinary sector. More specifically, the infusion or the decoction was given to cattle in case of digestion problems or when it was obvious that the animal was not feeling well, even when the specific cause of illness was not clear. Finally, the species was reported 2 times in the food sector (the infusion drunk just as pleasant herbal tea) and 2 times in the agropastoral sector (as animal feed for rabbits and swine).

### 2.2. The Identification Process of the Species During the Interviews

The first reports of the species, known by its dialectal name of ‘*Erba del Vinil*’ or ’*Erba del Venil*’, came out during some of the first interviews in late winter, early spring 2022. Since it was still too early to be able to observe the species in the field, first, we asked the informants to describe the plant as they remembered it. The descriptions obtained during this first phase of the process, however, seemed to be hazy and clashed with one another, especially due to the fact that it was remembered almost only by the elders, the majority of whom had not collected it for decades: ‘I remember it has yellow flowers, but I cannot describe them now’, ‘Honestly, I have never seen the flowers, it has only leaves arranged together at the base’, and again, ‘Yes, it has flowers, but they are not yellow! They are green-brownish, and they stick to the clothes’.

The second fortuitous step of the identification involved a chat with a local expert, who was not included among the informants because of their background as a herbalist. During this exchange, they recalled being told many years before by some elders of the valley that locals would collect a plant called ‘*Erba del Vinil*’ and, after observation, they suspected it could be agrimony.

The third step was to ask one of the informants of the valley to take and send pictures of the species, even if only of the basal rosette during spring. This was followed by the fourth step, which was to be accompanied in the field by said informant during the summer flowering of the species for the direct observation and collection of plant material and samples. During the outing, pictures of the species, which in fact turned out to be *A. eupatoria*, were taken as well. The last step was the determination of the subspecies *A. eupatoria* subsp. *eupatoria*, performed in the laboratory, as described in Section 3.2.

**Figure 2:**
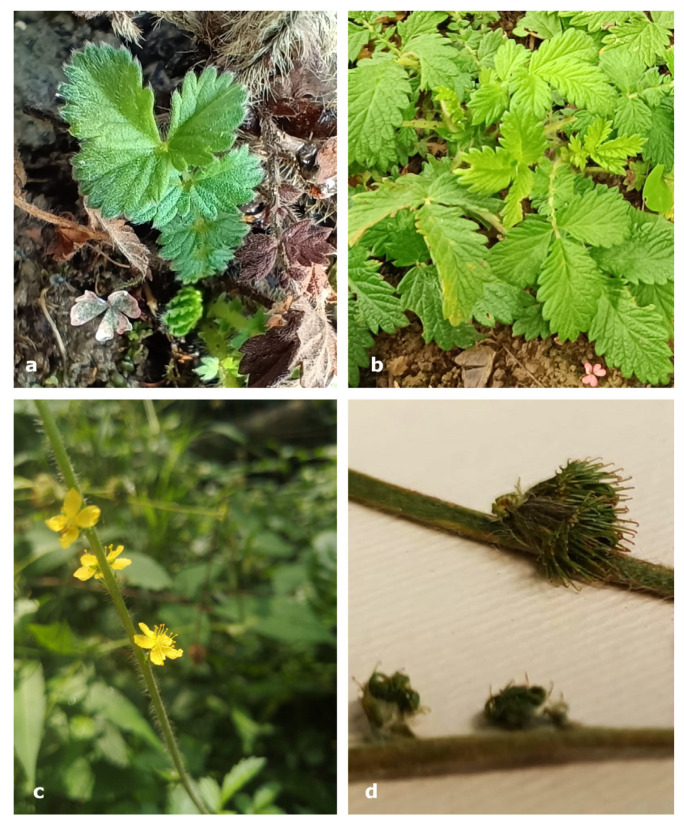
Pictures of *A. eupatoria* taken during the field work at different phenological stages. (**a**,**b**) leaves of the basal rosette; (**c**) inflorescences during anthesis; (**d**) fruits.

After the identification of the species (Figure 2), the descriptions and tales of the informants were finally clear. In fact, some of the locals collected and used the leaves of *A. eupatoria*, while others preferred the aerial parts. Additionally, the collection time in this valley ranged from early April to early October, which allowed them to observe the species at various phenological stages, thus providing a justification for their initial conflictual descriptions.

### 2.3. Morphological Investigation

#### 2.3.1. Glandular and Non-Glandular Indumenta

**Figure 3:**
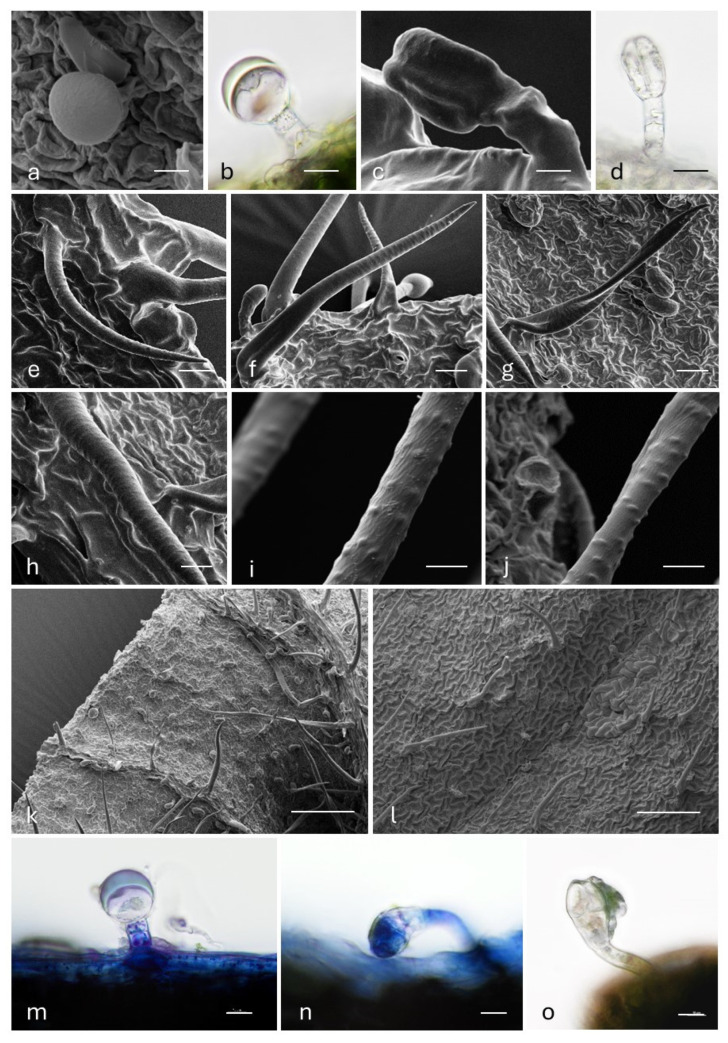
(**a**–**d**) SEM and LM micrographs showing the glandular trichome morphotypes observed on the leaf surfaces of *A. eupatoria* subsp. *eupatoria*: (**a**,**b**) the capitate with a 1-celled rounded head and 2 (3)-celled stalk; (**c**,**d**) the capitate with a 2-celled cylindrical head and 2 (3)-celled stalk. (**e**–**j**) SEM micrographs showing the non-glandular trichomes morphotypes observed on the leaf surfaces of *A. eupatoria* subsp. *eupatoria*: (**e**) curved trichomes; (**f**) straight trichomes; (**g**) straight trichomes with a filamentous and twisted appearance; (**h**) transversely elongated papillae on the cuticle of the non-glandular trichomes; (**i**,**j**) slightly echinate or verucate ornamentations on the cuticle of the non-glandular trichomes. (**k**,**l**) SEM micrographs showing the distribution pattern of the non-glandular and glandular indumenta on the leaves of *A. eupatoria* subsp. *eupatoria*: (**k**) leaf abaxial surface; (**l**) leaf adaxial surface. (**m**–**o**) LM micrographs showing the histochemical results on the secretory materials of the glandular trichomes of *A. eupatoria* subsp. *eupatoria*: (**m**) rounded capitate, Nadi reagent; (**n**) cylindrical capitate, Nadi reagent; (**o**) cylindrical capitate, Ferric Trichloride test. Scale bars: 5 μm (**a**–**c**,**m**–**o**); 10 μm (**d**–**j**); 100 μm (**k**,**l**).

The leaf surfaces of *A. eupatoria* subsp. *eupatoria* were characterized by the occurrence of both glandular and non-glandular trichomes (Figure 3a–l). With regard to the glandular *indumentum*, we observed two trichomes morphotypes, a capitate with a one-celled rounded head and two (three)-celled stalk (Figure 3a,b) and a capitate with a two-celled cylindrical head and two (three)-celled stalk (Figure 3c,d), corresponding, respectively, to Type I and Type II hairs, described in [10] in other subspecies of different origin and in several members of the tribe *Agrimoniae*. Our results were inconsistent with the literature data, since previous authors did not observe the occurrence of a glandular *indumentum* in the nominal subspecies. As a matter of fact, they exclusively cited the rounded capitate (Type I) for *A. eupatoria* subsp. *grandis* and reported the cylindrical capitate (Type II) even as lacking in the whole genus.

Concerning the non-glandular *indumentum*, we observed two types of hairs, already described in the nominal species [10]: curved trichomes (Figure 3e) and straight trichomes forming variable angles with respect to the epidermis (Figure 3f). The latter have variable lengths and may sometimes appear as filamentous and twisted (Figure 3g) and is described in Faghir et al. [10] as the third type of non-glandular trichome.

Furthermore, the cuticular ornamentations were characterized by the presence of transversely elongated papillae (Figure 3f,h) reported as typical of the nominal subspecies [10]. Slightly echinate or verucate surfaces were sporadically observed (Figure 3i,j), while they were reported only in other subspecies [10].

Regarding the distribution pattern, the two capitate morphotypes were located on both leaf epidermises, occurring on the whole laminas, with greater density on the lower one (Figure 3k,l). Along the edges, we predominantly observed the cylindrical morphotype. The non-glandular hairs occurred on both leaf surfaces as well, with a preferential distribution on the vein system of the lower epidermis (Figure 3k,l).

Overall, our results suggest that the nomenclature used in [10] to describe the secretory structures needs revision. Additionally, considering the discrepancies with the work of these authors, their suggestion of using these micromorphological features as a tool to discriminate different subspecies of *A. eupatoria* should probably be reconsidered.

#### 2.3.2. Histochemistry

The results of the histochemical investigation, which represent an element of novelty in the literature, are reported in Table 2 and Figure 3m–o. Both capitate morphotypes were seen to be responsible for the production of terpenic substances (Figure 3m,n; Table 2), which were stored in the subcuticular space and released after cuticle rupture. The cylindrical capitates also positively responded to Ferric Trichloride staining (Figure 3o; Table 2), thus highlighting the presence of polyphenols. Alcian Blue and Ruthenium Red staining gave negative responses, indicating the absence of acid polysaccharides and muco-polysaccharides in the secretory material.

### 2.4. Characterization of the Polar Extracts

#### 2.4.1. The Identification of Phenolic Compounds in the Different Aqueous Extracts from Agrimony

The mass spectrometry analysis of the extracts obtained from agrimony samples allowed for the identification and quantification of 34 molecules, belonging to different subclasses of phenolic compounds. Detailed information about the different molecules is reported in Table 3. The analysis revealed the presence of three simple phenols, representing the 9% of the total number of the molecules, eight phenolic acid derivatives (24%), 11 molecules derived from flavonol aglycones (32%), three flavone derivatives (9%), eight flavan-3-ols (24%), and pedunculagin, which belongs to the ellagitannin subclass (3%). Simple phenols were identified by MS/MS profiles and retention time (RT), according to the literature [31]. Among others, several groups of isomeric molecules were found. When possible, their discrimination was conducted on the basis of the literature and/or we performed dedicated analyses with standards.

The chromatographic peaks **5**, **6**, **7**, **11**, and **14** were assigned to caffeoyl derivatives due to the presence in the MS/MS profile of the fragments at 179, 161, and 135 *m*/*z* [32]. The isomers **6**, **7,** and **11** were discriminated according to Jaiswal et al. [33]. The two 6-caffeoylglucose molecules (**7** and **11**) were characterized by the fragments at 281, 251, and 221 *m*/*z*, and the α/β anomers differed in RT. α-1-caffeoylglucose (**6**) was assigned on the basis of its RT and fragmentation profile, which lacks a fragment at 203 *m*/*z* typical of the β anomer. Caffeoylquinic acid isomers were distinguished by RTs and MS/MS profiles [31,34]. Neochlorogenic acid (**5**) showed a major base peak at 191 *m*/*z*, which corresponds to the quinic acid moiety, and eluted earlier than the chlorogenic acid standard (RT = 7.06 ± 0.14 min), while 4-O-caffeoylquinic acid (**14**) eluted later and was characterized by the major base peak at 173 *m*/*z*, corresponding to a water loss from 191 *m*/*z*.

Molecules containing a coumaric moiety gave a distinctive fragment at 163 *m*/*z* (**8**, **13**, and **19**) [31]. In the profile of the coumaroyl acid hexoside (**13**), the 145 *m*/*z* probably derived from the further loss of a water molecule. The isomers of coumaroylquinic acid showed fragments originated from the quinic acid moiety (191 and 173 *m*/*z*) and were discriminated on the basis of the RTs. Isomer 4-O-*p*-coumaroylquinic acid was excluded using the standard (RT = 9.85 ± 0.01). Moreover, 3-O-*p*-coumaroylquinic acid (**8**) eluted earlier than 5-O-*p*-coumaroylquinic (**19**), and the two molecules showed fragments at 119 and 173 *m*/*z*, respectively, as previously described [33].

The flavonol derivatives were identified due to the presence in the MS/MS profile of the ions corresponding to quercetin (301 and 300 *m*/*z*) or to kaempferol (285 and 284 *m*/*z*). For each aglycone, the first and the second ion derive from the loss of the sugar moiety via the heterolytic and homolytic bond cleavage, respectively [35]. Hyperoside (**25**, i.e., quercetin 3-galactoside) and quercetin 3-glucoside (**26**) were assigned using standards. Similarly, peak **24** was assigned to rutin. Meanwhile, peak **22** was tentatively assigned to quercetin 3-O-rhamnoside 7-O-glucoside, considering that the literature suggests that other known isomers eluate after rutin [36,37]. Quercetin O-galloyl-hexoside (**21**), kaempferol O-glucoside (**29**), and kaempferol 3-O-rutinoside (**28**) showed MS/MS profiles already described [13,31]. The two precursor ions at 593 *m*/*z* coincided with tiliroside (kaempferol 3-O-coumarylglucoside), and they were assigned to the *trans*- and *cis*-isomers (**31** and **32**) on the basis of the RTs [38]. Similarly, the precursor ions at 635 *m*/*z* correspond to acetyl-tiliroside, a molecule characterized in agrimony for the first time in 2010 by Lee and their group, through NMR and HPLC, showing higher hydrophobicity than tiliroside [16]. Interestingly, we found two isomers (**33** and **34**) that could be interpreted as *trans*- and *cis*-isomers. To the best of our knowledge, for now, there is no information in the literature, nor a chemical standard, that can be used to validate this hypothesis. The two isomers **23** and **30** and peak **27** were assigned to vitexin, apigenin 7-O-glucoside, and Luteolin 7-O-glucoside, respectively, considering that the RTs and MS/MS profiles were consistent with the results obtained in agrimony by Santos and their group [13].

All of the flavan-3-ol derivatives showed major signals in the MS/MS profiles comparable to those previously reported [9]. In detail, the precursor ion at 1153 *m*/*z* and its fragment at 863 *m*/*z* correspond to a procyanidin tetramer (**16**). The MS/MS profiles and RTs of Catechin (**12**), procyanidin B2 (**17**), and procyanidin C1 (**20**) were verified through chemical standards. Epicatechin (**18**), procyanidin dimers B1 (**9**) and B3 (**10**), and procyanidin trimer C2 (**15**) were assigned on the basis of their relative RTs compared to the standards and according to Rzeppa and colleagues [39].

Finally, the ellagitannin pedunculagin (**4**) was assigned on the basis of its major fragment ion, according to the literature [14].

All the chromatograms can be found in the Supplementary Material file as Figures S1–S7.

**Table 3:** Identification of compounds by LC-ESI-MS/MS. N: peak number; RT: retention time range (n = 3); [M-H]^−^: molecular ion detected in negative mode; *m*/*z*: mass/charge; MS/MS fragment profile: *m*/*z* of the fragment ions (the average relative abundances of each fragment with a cut-off of 10% are reported in brackets) (n = 3); CE: collision energy (V); S: comparison with standard; Ref.: references.

N	Compound	Formula	RT (min)	[M-H]^−^ (*m*/*z*)	MS/MS Fragment Profile	CE	S	Ref.
Simple phenols								
1	Gallic acid	C_7_H_6_O_5_	1.73 ± 0.01	169.01	169 (10), 125 (100)	15		[31]
2	Hydroxybenzoic acid	C_7_H_6_O_3_	2.07 ± 0.02	137.04	93 (100)	15		
3	Vanillic acid	C_8_H_8_O_4_	3.20 ± 0.03	167.04	152 (100), 108 (80)	15		
Phenolic acid derivatives								
6	α-1-caffeoylglucose	C_15_H_18_O_9_	4.96 ± 0.02	341.09	341 (11), 179 (43), 161 (100), 162 (12)	15		[32,40]
7	β-6-caffeoylglucose	C_15_H_18_O_9_	5.55 ± 0.03	341.09	281 (92), 251 (36), 221 (46), 179 (100), 161 (27)	15		
11	α-6-caffeoylglucose	C_15_H_18_O_9_	6.43 ± 0.03	341.09	281 (63), 251 (26), 221 (36), 179 (100), 161 (32), 135 (17)	15		
5	Neochlorogenic acid	C_16_H_18_O_9_	4.32 ± 0.03	353.09	353 (15), 191 (100), 179 (75), 135 (15)	15	S	[31,34]
14	4-O-caffeoylquinic acid	C_16_H_18_O_9_	7.45 ± 0.07	353.09	191 (36), 179 (76), 173 (100), 135 (18)	15	S	
13	Coumaroyl acid hexoside	C_15_H_18_O_8_	6.83 ± 0.03	325.09	163 (14), 145 (100)	15		[31]
8	3-O-*p*-Coumaroylquinic acid	C_16_H_18_O_8_	5.64 ± 0.03	337.09	191 (29), 163 (100), 119 (14)	15		[31,33]
19	5-O-*p*-Coumaroylqunic acid	C_16_H_18_O_8_	9.50 ± 0.04	337.09	173 (100), 163 (21)	15		
Flavonol derivatives								
25	Hyperoside	C_21_H_20_O_12_	14.02 ± 0.03	463.09	301 (32), 300 (100), 271 (15)	30	S	[31,35]
26	Quercetin 3-glucoside	C_21_H_20_O_12_	14.38 ± 0.04	463.09	301 (35), 300 (100), 271 (15)	30	S	
22	Quercetin 3-O-rhamnoside 7-O-glucoside	C_27_H_30_O_16_	13.60 ± 0.10	609.15	609 (64), 301 (44), 300 (100)	30		[35]
24	Rutin	C_27_H_30_O_16_	13.89 ± 0.03	609.15	609 (64), 301 (66), 300 (100)	30	S	[31,35]
21	Quercetin O-galloyl-hexoside	C_28_H_24_O_16_	12.92 ± 0.18	615.10	615 (83), 464 (25), 463 (100), 301 (23), 300 (25)	25		[13]
29	Kaempferol O-glucoside	C_21_H_20_O_11_	16.27 ± 0.03	447.09	447 (11), 285 (88), 284 (96), 255 (30), 227 (18)	30		
28	Kaempferol 3-O-rutinoside	C_27_H_30_O_15_	15.74 ± 0.03	593.15	593 (35), 285 (100), 284 (50)	30		[31]
31	*trans*-tiliroside	C_30_H_26_O_13_	22.88 ± 0.03	593.13	593 (24), 285 (100), 284 (67)	30		[38]
32	*cis*-tiliroside	C_30_H_26_O_13_	23.55 ± 0.02	593.13	593 (23), 285 (100), 284 (67)	30		
33	Acetyl-tiliroside (1)	C_32_H_28_O_14_	26.98 ± 0.03	635.14	635 (13), 575 (14) 285 (37), 284 (100), 283 (17), 255 (10)	30		-
34	Acetyl-tiliroside (2)	C_32_H_28_O_14_	27.64 ± 0.02	635.14	635 (26), 285 (100), 284 (71)	30		-
Flavone derivatives								
23	Vitexin	C_21_H_20_O_10_	13.77 ± 0.03	431.10	431 (100), 341 (36), 311 (79)	15		[13]
30	Apigenin 7-O-glucoside	C_21_H_20_O_10_	16.80 ± 0.04	431.10	431 (21), 269 (36), 268 (100)	30		
27	Luteolin 7-O-glucoside	C_21_H_20_O_11_	14.59 ± 0.06	447.09	285 (100), 284 (45)	30		
Flavan-3-ol derivatives								
9	Procyanidin B1	C_30_H_26_O_12_	5.90 ± 0.03	577.13	577 (100), 451 (37), 425 (84), 407 (75), 299 (10), 289 (79), 287 (22), 161 (10), 125 (41)	15		[13]
10	Procyanidin B3	C_30_H_26_O_12_	6.21 ± 0.06	577.13	577 (100), 451 (32), 425 (90), 407 (68), 299 (11), 289 (68), 287 (20), 161 (10), 125 (36)	15		
12	Catechin	C_15_H_14_O_6_	6.70 ± 0.04	289.07	289 (100), 245 (74), 205 (30), 203 (30), 179 (21), 165 (10), 151 (13), 137 (16), 125 (28), 123 (11), 109 (27)	15	S	
15	Procyanidin C2	C_45_H_38_O_18_	7.60 ± 0.05	865.20	865 (100), 713 (10), 577 (15), 575 (16)	15		
16	Procyanidin tetramer	C_21_H_20_O_11_	7.94 ± 0.04	1153.23	1153 (93), 863 (25), 577 (53), 575 (73), 287 (39)	25		
17	Procyanidin B2	C_30_H_26_O_12_	8.54 ± 0.27	577.13	577 (97), 451 (37), 425 (99), 407 (80), 289 (78), 287 (24), 125 (43)	15	S	
18	Epicatechin	C_15_H_14_O_6_	9.25 ± 0.05	289.07	289 (100), 245 (72), 205 (30), 203 (29), 179 (22), 165 (11), 151 (14), 137 (16), 125 (29), 123 (11), 109 (26)	15		
20	Procyanidin C1	C_45_H_38_O_18_	10.92 ± 0.07	865.20	865 (100), 713 (12), 577 (17), 575 (17)	15	S	
Ellagitannins								
4	Pedunculagin	C_34_H_24022_	3.49 ± 0.02	783.07	783 (100), 301 (45)	25		[14]

#### 2.4.2. Qualitative and Quantitative Comparison Among the Phenolic Composition of the Different Extracts

First, concerning the percentage yields of the aqueous extractions (the total quantity of liophylized extract obtained in relation to the total quantity of source dried plant material), the results were as follows: AgrEpi35 = 3.32 ± 1.50%; AgrInfL = 9.62 ± 0.27%; AgrDecL = 11.71 ± 0.90%; AgrInfP = 8.62 ± 0.98%; AgrDecP = 11.28 ± 1.05%.

Among the five extracts, the epicuticular extract contained only low amounts of Luteolin 7-O-glucoside (**27**), Catechin (**12**) and Epicatechin (**18**). A comparison of different extraction procedures (E) and plant materials (M) allowed for the observation of interesting differences (Table 4).

Considering the total amounts of the identified molecules, the statistical analysis showed an interaction between E and M. In detail, the decoction of leaves assured a major extraction yield, higher than all the other preparations. This result might be derived from two concomitant aspects. Firstly, the leaves represent a plant sample prevalently composed by metabolic active tissues, probably rich in bioactive molecules. On the contrary, the entire plant could also contain less active tissues, i.e., vascular and/or lignified ones. Secondly, it is likely that the stronger manipulation of the sample during decoction is able to increase the release of molecules from the tissues.

Overall, the flavone, flavan-3-ol, and flavonol derivatives represented the major abundant subclasses, and could be interpreted as characteristic of the agrimony extracts. However, E and M differently affected the extraction of subclasses and also individual molecules. Simple phenols were mainly affected by E, being more abundant in decoctions. This was particularly evident for gallic acid (**1**). Otherwise, phenolic acid derivatives showed higher abundance in the extracts obtained from the leaves, such as Caffeoylglucose isomers (**6**, **7**, and **11**) and neochlorogenic acid (**5**). However, two of the most abundant molecules of this subclass, coumaroyl acid hexoside (**13**) and 5-O-*p*-coumaroylquinic acid (**19**), were significantly higher only in leaf decoctions. The subclass of flavonols was particularly influenced by the interaction between the two factors (I, i.e., ExM). Once again, leaf decoction was the procedure that assured major extraction yields, considering the whole subclass but also individual molecules, such as Quercetin 3-glucoside (**26**), rutin (**24**), and tiliroside derivatives (**31**–**34**). Some exceptions concerned interesting molecules, such as hyperoside (**25**), whose extraction yield decreased only in the leaf infusion, and kaempferol O-glucoside (**29**), only detectable in the whole plant extracts. Flavones constituted the major subclass, although it was composed by only three molecules. The subclass was independently affected by E and M, and overall, the molecules were more abundant in the leaves and, especially, in decoctions. In detail, vitexin (**23**), Apigenin 7-O-glucoside (**30**) and Luteolin 7-O-glucoside (**27**) represented 4.4%, 7.6%, and 43.4% of the total amount of the identified molecules in the leaf decoction, respectively. The flavan-3-ol subclass was independently influenced by E and M, but it showed the highest variability. Besides Catechin (**12**) and Epicatechin (**18**), which were the most abundant, this class comprised more complex molecules, such as dimers (**9**, **10**, and **17**), trimers (**15** and **20**), and a tetramer (**16**). Firstly, it is noteworthy that these molecules are widely distributed in all plant tissues. Secondly, their physical–chemical diversity might justify some of the observed variability. Lastly, the analysis highlighted the presence of low amounts of pedunculagin (**4**) in the extracts from the whole plant.

**Figure 4:**
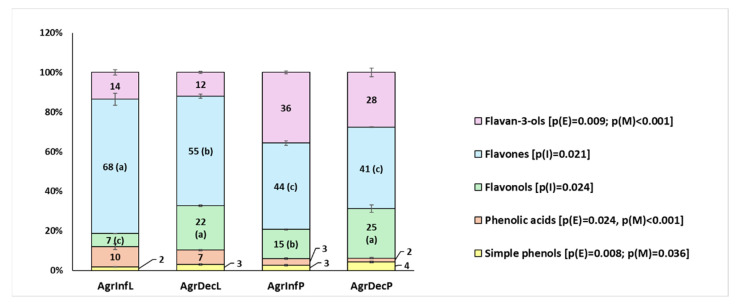
Relative percentages of the major phenol subclasses in the agrimony extracts. Values are expressed as average percentages ± SE (n = 3). Different letters indicate significant differences. Detailed information regarding the significant effect is indicated in the legend. *p*(E): *p* value of the effect of the extraction method; *p*(M): *p* value of the effect of the plant material; *p*(I): *p* value of the interaction. To improve clarity, the ellagitannin subclass is not included since it represents less than 0.2% of the total amount.

Additionally, by expressing the amount of each subclass as its relative percentage of the total allowed us to highlight several differences in composition among the extracts (Figure 4). Such differences could confer different bioactive properties to the extracts and could be somehow related to their different traditional modes of use. In particular, the use of agrimony leaf allowed us to obtain extracts particularly rich in flavones, while the use of the whole plant resulted in an enrichment in flavan-3-ols, especially through infusion. This last aspect might be related to the thermolability of complex Catechin derivatives. Additionally, the stronger heat treatment during decoction improved the extraction of flavonols. Finally, phenolic acids and simple phenols were better extracted from leaves or by decoction, respectively. This could be due to their abundances in the tissues and also to their solubility.

Considering the published literature, while there were no epicuticular aqueous extracts, a couple of comparable tissue aqueous extracts (infusions of the aerial parts) could be found [18,19]. Overall, our results were consistent with those previously reported, especially considering the most abundant molecules characteristic of agrimony extracts (i.e., **10**, **12**, **14**, **23**, **25**–**27**, **30**).

Concerning the activity of these compounds at the skin level, especially in the wound-healing process, some interesting results could be found in the literature. Specifically, Catechin (**12**) and Epicatechin (**18**) were, along with Luteolin 7-O-glucoside (**27**), the only compounds common to all five extracts. While no works were found on **27**, Monika and their group investigated the anti-inflammatory activity of two enriched extracts (one containing 83% of Catechin from *Camellia sinensis* (L.) Kuntze and the other 17% of Epicatechin from *Acacia* spp.) in acute and chronic wound fibroblasts, detecting a reduction in the levels of TGF-β and TNF-α at doses of 100 μg/mL [41]. The potential wound-healing activity was also investigated for hyperoside (**25**) and vitexin (**23**), two compounds found in both decoctions and infusions of agrimony [42,43]. Moreover, extracts rich in gallic acid (**1;** found only in the decoctions), rutin (**24**; in both infusions and decoctions), and Kaempferol-3-O-glucoside (**29**; only in the aerial parts) were shown to have both antimicrobial (against *S. aureus*) and anti-inflammatory activities [44]. Finally, the topical anti-inflammatory activity was also demonstrated for various procyanidins, such as Procyanidin B3 (**10**, [45]), especially present in our two decoctions.

### 2.5. Characterization of the Non-Polar Extracts

#### Volatile Organic Compounds Spontaneously Emitted by Fresh Leaves

The volatile organic compound (VOC) analysis in the fresh leaves of *A. eupatoria* identified through Gas Chromatography–Mass Spectrometry (GC-MS) revealed a complex and diverse profile, with each compound contributing to the overall volatile content (Table 5). This study shows that the total VOCs detected constitute 100% of the sample, representing the plant’s aromatic profile. Monoterpene hydrocarbons (MHs) dominated the VOC composition, constituting 92.60% of the total volatile content. Under the applied experimental conditions and within the context of the fiber’s selectivity, (+)-α-Pinene (**2**) was the most abundant compound within this group, accounting for a relative abundance of 81.39% of the total VOCs. This overwhelming presence would suggest (+)-α-Pinene to be the primary contributor to the aromatic profile of *A. eupatoria* fresh leaves. Other notable monoterpenes included (−)-Limonene (**11**) and β-Thujene (**6**), which constituted 5.36% and 3.92% of the whole profile, respectively. Although present in smaller amounts than (+)-α-Pinene, these compounds still significantly shape both the plant’s aromatic profile and the potential bioactivity. While less prominent than monoterpenes, sesquiterpene hydrocarbons (SHs) made a notable contribution of 2.36% to the overall VOC content. Within this subclass, (+)-Cuparene (**37**) stood out with a relative abundance of 1.06%. Other sesquiterpenes, such as α-Bisabolene (**36**; 0.15%) and α-Curcumene (**35**; 0.07%), though present in relatively minor quantities, added further complexity to the VOC profile. Despite their smaller quantities, oxygenated monoterpenes (OMs) made a potentially significant contribution of 0.24% to the total VOCs. Compounds like campholenic aldehyde (**18**; 0.05%) and isopinocamphone (**21**; 0.05%) represented this subclass. Oxygenated sesquiterpenes (OSs), despite their low concentrations, accounted for a noteworthy 0.45% of the total VOCs. Longiverbenone (**43**; 0.32%) and spathulenol (**41**; 0.05%) were the main representatives of this subclass. Finally, a diverse group of other compounds, including various benzoic acids, derivatives, and organosilicon compounds, contributed 4.35% to the total VOC content. Among these, 1-methyl-2-propan-2-ylbenzene (**10**) was particularly notable, with a relative abundance of 1.68%, while artemesia ketone (**14**) accounted for 1.39%. This diverse and complex blend of volatiles not only defines the aromatic properties of the plant but may also have implications for its medicinal and ecological roles.

While we were not able to find VOC profiles of *A. eupatoria* in the literature, a couple of essential oil profiles (EOs) could be found [20,46]. Specifically, in 2013, Feng and their group investigated the EOs of leaves and roots of the species, detecting a total of 68 compounds [20]. The profile was dominated by the oxygenated sesquiterpene cedrol, which accounted for 14.37% of the total profile, followed by the monoterpene hydrocarbon α-Pinene (8.31% of the total). The oxygenated monocarbons linalool and α-Terpineol further enriched the profile with 5.72% and 4.21%, respectively. Finally, compounds belonging to other diverse subclasses, such as the alkyl ketone 1-(2-Furyl)-1-hexanone (4.87%), completed the profile.

In 2023, Iqbal et al. investigated the EOs of aerial parts of *A. eupatoria*, detecting 24 total compounds [42]. The profile was clearly dominated by monoterpene hydrocarbons (71.3%) represented by α-Pinene (62.4% of the total profile). Sesquiterpenes followed (8.8% of the total), with α-Humulene and α-Farnesene accounting for 3.9% and 3.2%, respectively. Oxygenated monoterpenes (8.5%) were represented by Carvone, with 4.8%. Finally, compounds belonging to other subclasses, such as the diterpenoid Isophyllocladen, constituted 5.3%.

Concerning the biological activity at the skin level potentially ascribable to the compounds detected in the leaves’ VOC profile, promising results were found in the literature for the main compound (+)-α-Pinene (**2**). In fact, Salas-Oropeza and their group assessed the potential wound-healing activity of this monoterpene (and also of α-Phellandrene, which is present in our profile, even if only constituting 0.69% of the relative abundance) [43]. Both these compounds not only were shown to have low cytotoxicity at the tested doses but also to have wound-healing properties by enhancing collagen deposition, which in turn should speed up wound contraction and closure, and attracting fibroblasts to the wound site, thus improving re-epithelialization [47]. Additionally in 2022, (+)-α-Pinene was reported to both have significant antimicrobial activity against *Escherichia coli* (at the dose of 512 μg/mL) and improve the activity of conventional antibiotics against antibiotic-resistant bacteria [48]. While no studies were found concerning our second most abundant terpene, (−)-Limonene (**11**), and its potential activity at the skin level, some interesting works were published on its enantiomer, (+)-Limonene, in the literature. Specifically, this compound was studied for its wound-healing activity, which could be augmented both due to the improvement of wound contraction and re-epithelialization [49], and due to its anti-inflammatory activity, with lessened the production of pro-inflammatory cytokines at the wound site, associated with improved tissue regeneration and reduced neo-vascularization [50].

### 2.6. Antimicrobial Activity and Adhesion Modulation by Infusions and Decoctions of A. eupatoria

As the last step of this multidisciplinary investigation, we conducted preliminary tests to evaluate the potential biological activity of the four different aqueous extracts (infusions and decoctions, derived either from the aerial parts or the leaves, characterized during the previous phase, at Section 2.4.1).

**Figure 5:**
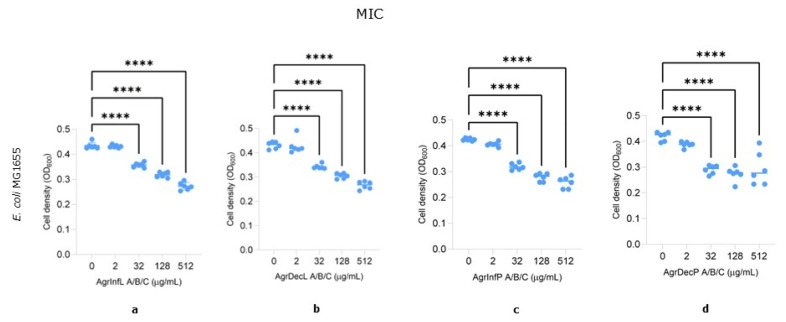
*A. eupatoria* effect on growth in *E. coli* MG1655. (**a**) AgrInfL (infusion leaves), (**b**) AgrDecL (decoction leaves), (**c**) AgrInfP (infusion aerial parts), (**d**) AgrDecP (decoction aerial parts). The control without compounds is YESCA or TSB. Results of at least 3 independent biological replicates are reported, with mean and SD displayed. ****, *p*-value < 0.0001, one-way ANOVA with Dunnett’s test for multiple comparisons.

First, we assessed the effect of the extracts on the bacterial growth of the Gram-negative enterobacteria *E. coli* MG1655, the pathogenic Gram-positive *S. aureus*, and the Gram-positive skin commensal *S. warneri*. We observed that all the extracts induced a significant, albeit mild, concentration-dependent reduction in *E. coli* MG1655 growth, starting from 32 µg/mL and with an average decrease of ca. 40% (range 35–45%) at the highest concentration tested (512 µg/mL) (Figure 5a–d). On the contrary, no significant effect was observed when the antimicrobial activity was evaluated using *S. aureus* and *S. warneri* (Figure S6, Supplementary Materials).

**Figure 6:**
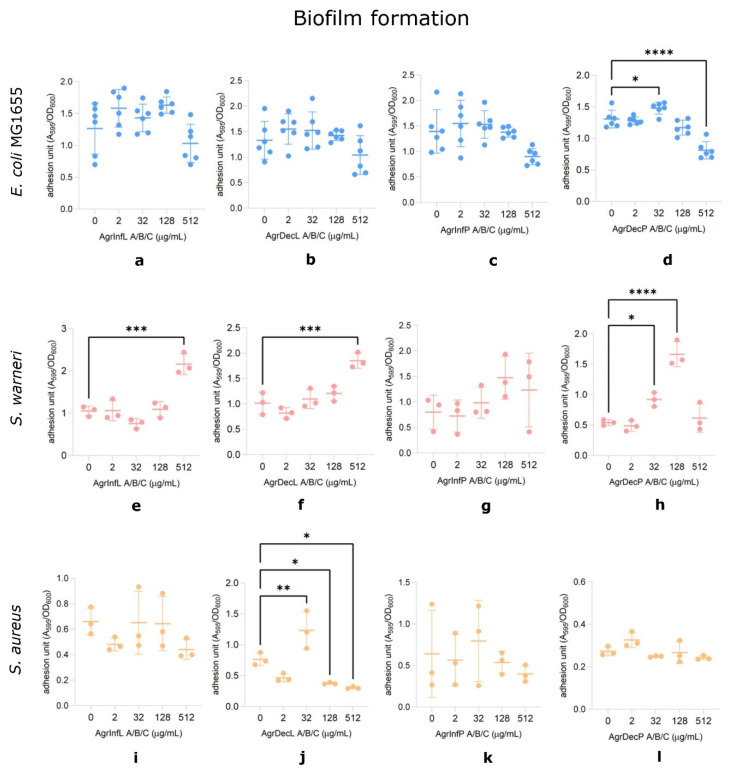
*A. eupatoria* effect on biofilm formation in *E. coli* MG1655, *S. aureus*, and *S. warneri*. (**a**,**e**,**i**) AgrInfL (infusion leaves), (**b**,**f**,**j**) AgrDecL (decoction leaves), (**c**,**g**,**k**) AgrInfP (infusion aerial parts), (**d**,**h**,**l**) AgrDecP (decoction aerial parts). The control without compounds is YESCA or TSB. Results of at least 3 independent biological replicates are reported, with mean and SD displayed. *, *p*-value < 0.05; **, *p*-value < 0.01; ***, *p*-value < 0.001; ****, *p*-value < 0.0001, one-way ANOVA with Dunnett’s test for multiple comparisons.

Second, we investigated the modulating activity of the same extracts on the adhesion capacity of the bacteria. Biofilm formation highly contributes to bacterial pathogenicity during infections, allowing the colonization of tissues and conferring protection against both the host immune system and antimicrobial treatments [51]. In contrast to bacterial growth, the extracts had no significant effect on the bacterial adhesion of the *E. coli* MG1655 strain, except for the AgrDecP extract, which showed a statistically significant reduction of approximately 40% at the highest concentration (512 µg/mL; Figure 6d). The effect on *Staphylococci* was more complex. Similar to *E. coli*, we could not observe any major impact on *S. aureus* apart from the AgrDecL extract, which caused an increase of about 40% in biofilm formation at 32 µg/mL (Figure 6j) but an overall minor inhibitory effect at higher concentrations (128 µg/mL and 512 µg/mL) (Figure 6j). In contrast, *S. warneri* biofilm formation was stimulated up to 1.9 times when the cultures were added with the AgrInfL and AgrDecL samples at 512 µg/mL, while for the AgrDecP extract, we observed an overall stimulatory effect at 32 µg/mL and 128 µg/mL, which was not maintained at higher concentrations (512 µg/mL) (Figure 6e–h).

Overall, our results would suggest that extracts of *A. eupatoria* are able to exhibit antibacterial activity against *E. coli* MG1655, affecting its growth and resulting in a minor inhibition of biofilm formation in *S. aureus*.

Concerning the comparable antibacterial activity shown by both the infusions and decoctions against *E. coli*, interesting observations can be made about Catechin (**12**), one of the most abundant compounds of all four extracts. In fact, Catechin seems able to perform antibacterial activity against *E. coli* due to its damaging activity at bacterial membrane level, possibly through oxidative mechanisms [52]. Moreover, previous studies have already reported the antimicrobial activity of *A. eupatoria* alcoholic extracts [4,21,23] against *S. aureus*. For example, extracts rich in gallic acid (**1**; found only in the decoctions), rutin (**24**; in both infusions and decoctions), and Kaempferol-3-O-glucoside (**29**; only in the aerial parts) were shown to have both antimicrobial activity against *S. aureus*) [40].

Actually, our experiments did not confirm the activity against *S. aureus*, likely due to various extraction methods and differences in the concentration range tested. However, we were able to verify the biological activity of the plant extracts against bacteria, as well as their ability to enhance biofilm formation in *S. warneri*. These results were surprising, especially considering the presence of quercetin and kaempferol derivatives in the extracts, known biofilm inhibitors [53,54,55]. This observation probably reflects the chemical complexity of the extracts, possibly suggesting the presence of molecules that might have opposite effects on *S. warneri* while not affecting the inhibition of biofilm formation in *E. coli* MG1655.

Overall, considering our findings on the partial but significant inhibitory effect on *E. coli* growth and the significant enhancing of *S. warneri* biofilm formation, we could hypothesize that the traditional application of external compresses of *A. eupatoria* aqueous extracts might be at least partially explained by its ability to restrain skin colonization by pathogenic bacteria while promoting it for the commensal skin microbiota. In fact, coagulase-negative staphylococci, such as *S. warneri*, directly compete with pathogens via different mechanisms [56] to maintain skin homeostasis and colonization resistance [57].

## 3. Materials and Methods

### 3.1. Area of Study

**Figure 7:**
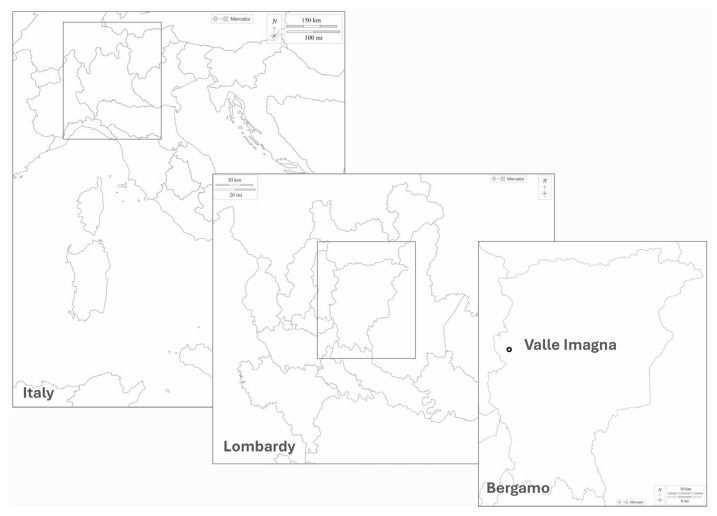
Valle Imagna is a pre-alpine valley of the province of Bergamo, Lombardy region, Northern Italy. Map obtained from the original maps at https://d-maps.com/m/europa/italia/italie_it/italie_it20.pdf (accessed on 20 January 2025), https://d-maps.com/m/europa/italia/lombardie/lombardie16.pdf (accessed on 20 January 2025), https://d-maps.com/m/europa/italia/bergamo/bergamo06.pdf (accessed on 20 January 2025).

Valle Imagna is a pre-alpine valley the western Orobic Prealps in the province of Bergamo, Lombardy, the North of Italy (Figure 7). The highest peak of the valley is Mount Resegone at 1875 m a.s.l. The area is named after Imagna, the main river of the valley. The valley is characterized by mild weather, with abundant rainfalls and limited temperature excursions [58].

During our survey, we focused on some of the municipalities of upper Valle Imagna, namely Berbenno (675 m a.s.l.), Brumano (911 m a.s.l.), Corna Imagna (736 m a.s.l.), Costa Valle Imagna (1014 m a.s.l.), Fuipiano Valle Imagna (1055 m a.s.l.), Rota d’Imagna (690 m a.s.l.), and Sant’Omobono Terme (427 m a.s.l.).

### 3.2. Ethnobotanical Survey, Data Archiving and Processing, Identification

The 109 open and semi-structured interviews, the data archiving, and the analysis of the datasets were conducted as described in Milani et al. because it was part of the same investigation [9].

Qualitative primary data concerning *A. eupatoria* were presented raw in their entirety for the first time, focusing on the emic vision of the respondents, following the current ethnobotanical literature [59].

The first identification of *A. eupatoria* was performed directly in the field by Professor Fico, Professor Giuliani, and Dr. Colombo PS, following [1]. *Exsiccata* of the species were produced, collected, coded, and deposited in the *Herbarium* of the Museum of Natural History of Milan (Code: MSNM 54011/54012; Figures S7 and S8, Supplementary Materials). Specifically, the collected samples were determined to belong to *A. eupatoria* subsp. *eupatoria* based on macroscopic diagnostic features: (*i*) *hypanthium*/crown length ratio > 1; (*ii*) erect external bristles; (*iii*) the presence of a basal rosette of leaves; (*iv*) a slightly pubescent stem (Table 6, [2,3]).

### 3.3. Plant Material

Concerning micromorphology, leaf samples from fresh plants of *A. eupatoria* were collected in Locatello (Valle Imagna, Bergamo) at around 650 m a.s.l. in August 2022 and July 2023 with the help of one of the key informants. We described the structure, the distribution pattern, and the histochemistry of trichomes on the leaves by means of Scanning Electron Microscopy (SEM), Light Microscopy (LM), and Fluorescence Microscopy (FM). For each examined plant part, at least ten replicates were analyzed to evaluate the variability level of the micromorphological features.

As for the extractions, leaves and flowered aerial parts (with the first portion of rhizome) of *A. eupatoria* were collected in Locatello (Valle Imagna, Bergamo) at around 650 m a.s.l. in July 2023 with the help of one of the key informants. The plant material was then dried at room temperature in a cool and aerate place.

For the analyses of the VOCs, plants were collected and potted in the field and then transported to the laboratory in order to maintain the integrity of the samples until the collection of the samples and the analysis.

### 3.4. Micromorphology and Histochemistry

#### 3.4.1. Scanning Electron Microscopy

The leaf samples were hand-prepared, fixed in an FAA solution (formaldehyde/acetic acid/ethanol 70% = 5:5:90) for 24 h, dehydrated in an ascending ethanol series up to the absolute value, and critical-point dried. The samples were mounted on aluminum stubs and gold-coated. Observations were performed under Zeiss^®^ EVO MA15 SEM operating at 10 kV at the Interdepartmental Center for Electron Microscopy and Microanalysis Services (M.E.M.A.) of the University of Florence (Florence, Italy).

#### 3.4.2. Light Microscopy and Fluorescence Microscopy

The micromorphological survey under LM and FM was performed on the fresh material. Sections ranging from 30 to 50 µm in thickness were obtained using a vibratome and/or a cryostat.

The following dyes were used [60]: Toluidine Blue as a general staining agent; Fluoral Yellow-88 for total lipids; Nile Red for neutral lipids; Nadi reagent for terpenes; Alcian Blue for mucopolysaccharides; Ruthenium Red for pectins; and Ferric Trichloride for polyphenols. Control procedures were carried out concurrently. Observations were made with a Leitz DM-RB Fluo optical microscope equipped with a Nikon digital camera.

### 3.5. Phytochemical Investigation

#### 3.5.1. Extractions

According to the traditional uses recorded during the field work, specifically the use of the leaves applied on wounded skin and of compresses with the infusion or decoction for the same purpose, we decided to focus on the epicuticular depositions on the leaves’ surfaces (both polar and apolar), as well as on infusion and decoction-like preparations.

For the epicuticular extracts, 2 g of whole leaves were carefully immersed in 200 mL of distilled water at 35 °C or in 200 mL of acetone for a total of 9 min. The extractions were performed in triplicate. The aqueous extracts were then filtered on filter paper, lyophilized, and subsequently stored at −20 °C. The acetonic extracts were filtered on filter paper, vaporized in Rotavapor^®^ (Heidolph, Schwabach, Germany), and subsequently stored in closed vials at room temperature.

Infusions were obtained by leaving 2 g of grounded leaves or 2 g of aerial parts for 8 min in 50 mL of distilled water at boiling point. For the decoctions, 2 g of grounded leaves or 2 g aerial parts were covered with 50 mL of distilled water at room temperature. The water was then heated until boiling point and left boiling for 8 min. Infusions and decoctions were then filtered on filter paper, left cooling, lyophilized, and subsequently stored at −20 °C. Every type of extract was produced in triplicate.

#### 3.5.2. LC-ESI-MS/MS Analysis on Aqueous Extracts

The analysis of the phenolic composition was carried out on the aqueous extracts obtained as described in Section 3.5.1. Lyophilized samples were resuspended with 50% (*v*/*v*) methanol (MOH) and 1% (*v*/*v*) formic acid (FA), filtered by sterilized polyvinylidene difluoride membrane (0.45 µm), and appropriately diluted to a final concentration of 250 ng/µL in 5% (*v*/*v*) MOH and 0.1% (*v*/*v*) FA. In total, 5 µL of each sample was analyzed by an Agilent Technologies 1290 Series capillary pump coupled with a Jet Stream ESI source on a 6546 LC/Q-TOF mass spectrometer. LC runs were performed on an InfinityLab Poroshell 120 SB-C18 column (2.1 × 100 mm, 1.9 μm, Agilent Technologies, Leini, Italy) in acidic conditions [0.1% (*v*/*v*) FA], applying the following 30 min non-linear acetonitrile gradient: 0–1 min at 5% and 1–30 min to 35% with a flow rate of 300 µL min^−1^. The analysis was conducted in negative mode, setting the ESI source at 350 °C (sheath gas and gas temperature) and 3500 V and the fragmentor at 175 V. The data acquisition range was 50–2000 *m*/*z* (mass to charge) at 1 scan s^−1^. Chromatographic peak interpretation was performed with MassHunter Workstation software (version 10.0, Agilent Technologies). The search was conducted against a custom-made database consisting of the main phenolic compounds found in the literature, identifying the compounds as negative ions [M-H]^−^ with a tolerance of ±5 ppm. The compound assignment was refined by targeted MS/MS analyses with an isolation width of 4 *m*/*z* and retention time (RT) range of ±0.5 min. The targeted MS/MS analyses were performed applying a collision energy (CE) of 15 V, 25 V or 30 V. Isomer discriminations were performed using commercial standards and/or according to the literature. The quantification of compounds was conducted on MS spectra by extracting the EIC (extracted ion current) for [M-H]^−^, with a tolerance of 20 ppm, using adequate external calibration curves. In detail, ferulic acid was used to calibrate simple phenols; chlorogenic acid was used to calibrate phenolic acid derivatives; quercetin glucoside was used to calibrate quercetin, apigenin and luteolin derivatives; rutin was used to calibrate itself and kaempferol derivatives; and Catechin was used to calibrate itself, its derivatives, and ellagitannins.

#### 3.5.3. Head Space (HS)–Solid-Phase Micro Extraction (SPME)–GC-MS Analysis on VOCs

The plant’s volatile organic compounds (VOCs) were analyzed on fresh tissues through HS-SPME-GC-MS [61]. Plant material (1 g per replicate) was placed into 20 mL glass vials and incubated for 20 min at room temperature. Subsequently, SPME gray fiber (StableFlex, coated with divinylbenzene/carboxen on polydimethylsiloxane; 50/30 μm coating; Merk Life Science, Milan, Italy) were exposed to the VOCs released by the plants for 20 min to allow adsorption onto the fiber. The SPME fiber was then inserted into a gas chromatograph coupled with a mass spectrometer operating in splitless mode.

For the experiments, an Agilent GC-MS apparatus equipped with a MEGA 5MS capillary column (MEGA srl, Legnano, Milan, Italy) measuring 30 m × 0.25 mm × 0.25 µm (with a 10 m pre-column) was used. Helium was used as the carrier gas at a purity level of 6.0 and a 1 mL/min flow rate. The injector was settled at 200 °C, whereas the transfer line, the source, and the quadrupole were set at 250 °C, 300 °C, and 200 °C, respectively. The temperature program was set as follows: an isocratic hold at 45 °C for 7 min, followed by a temperature increase from 45 °C to 80 °C at a rate of 10 °C per minute, then from 80 °C to 200 °C at a rate of 20 °C per minute, and a final isocratic hold at 200 °C for 3 min.

Mass spectra were recorded in electronic impact (EI) mode at 70 eV, scanning at a range of 40–450 *m*/*z*, and the scan time was 0.2 s. The mass spectrometric solvent delay was settled as 2 min and a mixture of alkanes (C8–C30) was used for the Retention Index (RI) calculation and peak annotation.

### 3.6. Biological Activity

#### 3.6.1. Antimicrobial Activity

The antimicrobial activity of *A. eupatoria* aqueous extracts (the infusion of the leaves, AgrInfL; infusion of the whole aerial parts, AgrInfP; decoction of the leaves, AgrDecL; and decoction of the whole aerial parts, AgrDecP, as described at Section 3.5.1) was evaluated by measuring bacterial growth after 24 h either in the presence or absence of the plant extracts using a modified microdilution assay [62]. Concerning the *Escherichia coli* MG1655 strain, overnight cultures were obtained growing the bacterium for 24 h in YESCA medium (10 g/L casamino acids and 1 g/L yeast extract) at 30 °C in shaking conditions (200 rpm), while for *Staphylococcus warneri* and *S. aureus*, cultures were grown in TSB medium (17 g/L casein peptone, 3 g/L soy peptone, 5 g/L sodium chloride, 2.5 g/L dipotassium hydrogen phosphate and 2.5 g/L glucose) at 37 °C medium for 24 h.

*A. eupatoria* extracts were diluted in either YESCA or TSB medium to achieve 2× the final concentrations (512, 128, 32, and 2 µg/mL). A total of 100 µL of the diluted sample was aliquoted into a 96-well microplate, followed by 100 µL of the initial bacterial inoculum normalized at a final OD of 0.02 in YESCA (*E. coli*) or TSB (*S. warneri*; *S. aureus*). The optical density (OD) of the plate was measured at the initial time point (OD_600i_) and after 24 h of incubation at 30 °C for *E. coli* or 37 °C for *S. warneri* and *S. aureus* (OD_600f_).

The extracts’ inhibitory effect was assessed by calculating the difference in growth (ΔOD_600_ = OD_600f_ − OD_600i_). A ΔOD_600_ of 0.05 was considered a complete inhibition of growth.

#### 3.6.2. Modulation of the Adhesion

Biofilm formation assays were performed on the same 96-well microtiter plates utilized for antimicrobial activity determination, using the Crystal Violet (CV) staining method, as previously described [63]. The supernatant was transferred to a new plate and the OD_600_ was determined. The wells were washed with 100 µL of deionized water, and then 200 µL of 0.1% CV solution was added. After 15 min of incubation at room temperature, the CV was washed off with distilled water, and 200 µL of 96% ethanol was added to solubilize the residual stain. The absorbance at 595 nm (A_595_) was measured, and the adhesion unit was calculated as the ratio between A_595_ and OD_600_ (adhesion units = A_595_/OD_600_).

### 3.7. Statistical Analysis

For the LC-ESI-MS/MS, the statistical analyses were conducted on 3 biological replicates. Statistical analyses were performed using SigmaPlot version 15. The normality and the equal variance were checked using the Shapiro–Wilk test and the Brown–Forsythe test, respectively. The data were then compared using ANOVA, with the Holm–Sidak post hoc test (*p* < 0.05). In order to assess the effects of individual factors (the extraction method and plant material) and their interaction, two-way ANOVA was applied. Where the interaction between the two factors was significant (*p* < 0.05), all conditions were subjected to one-way ANOVA. On the contrary, where the interaction was not significant, the effect of an individual factor was evaluated separately.

Concerning the biological activity, the results of at least 3 independent biological replicates were obtained. Statistical analyses were performed using GraphPad Prism version 10. To compare means and standard deviations, one-way ANOVA was used with Dunnett’s test for multiple comparisons (*, *p* < 0.05; **, *p* < 0.01; *** *p* < 0.001; and ****, *p* < 0.0001).

## 4. Conclusions and Future Perspectives

The traditional use of ‘*Erba del Vinil*’, *A. eupatoria*, in Valle Imagna is undoubtedly almost faded, a memory that stays alive only in the mind of a few older inhabitants of the valley. However, the bond with this territory is not completely lost yet. This bond was what prompted us to investigate deeper, through a multi-step and multidisciplinary approach with the aim of validating or refuting the traditional use of the species as a topical anti-inflammatory and wound-healing agent, whether by applying the leaves to the wounds or washing them with compresses of infusions and decoctions.

The characterization of the polar extracts showed that decoction, especially for leaves, should result in a better extraction yield, compared to the other types of extraction. Specifically, if we take into account the compounds that are already known from the literature to be topically active, the decoction of the leaves was also the one with higher concentrations of these specific compounds, often followed by the decoction of aerial parts and the infusion of the leaves.

The epicuticular aqueous extract was the less rich of the five, albeit containing both Catechin and Epicatechin, which were among the most active molecules at the skin level. Moreover, the epicuticular profile must be considered supplementary to the VOC profile analyzed herein, which was shown to have other potentially active molecules in the case of skin wounds, i.e., antimicrobial or wound healing.

The preliminary biological activity screening, carried out on three bacterial species, highlighted interesting and promising results, although they need to be further investigated. Both the infusions and decoctions were shown to have antimicrobial activity and the capacity to modulate biofilm formation. Specifically, all four extracts were able to inhibit *E. coli* growth, while the infusion of aerial parts was the only one also able to inhibit *S. aureus*. The decoctions, leaves and aerial parts, respectively, were able to inhibit biofilm formation in *S. aureus* and *E. coli*. Finally, all the extracts (except for the infusion of aerial parts) positively modulated biofilm formation in *S. warneri*.

Further phytochemical and pharmacological analyses will be carried out on epicuticular extracts obtained from fresh leaves, not only aqueous but also acetonic, and on the essential oils from the aerial parts. In order to complete also the micromorphological and histochemical investigations, observations will be extended to the inflorescences and flowers.

Further in-depth analyses on the biological activity will be conducted not only on the already investigated extracts but also on the epicuticular extracts. Other than the antimicrobial and antibiofilm activities, we will also investigate the potential wound-healing properties of the extracts.

Finally, in the near future, we will organize an event dedicated to the population of Valle Imagna, with the aim to give all the information obtained in the field on *‘Erba del Vinil’* back to its inhabitants and to enrich it with all these scientific results, thus avoiding the loss of the traditional knowledge linked to this species.

## Figures and Tables

**Table 1:** Complete raw primary data reported for *A. eupatoria* in the medicinal sector of use.

Part of the Plant	Inform. Code	General Information	Preparation	Administration	Category of Use	Detailed Use
Plant *in toto*	COR006	80 years old; their knowledge comes from their family	Infusion	Compress	Anti-inflammatory, disinfectant, and wound healing	
			Infusion	Oral	Stomach ache	
	SAN005	86 yo. Mother of SAN006. Sometimes, they used it with *M. sylvestris.* Sometimes, flowers of *Sambucus nigra* L. or *Citrus limon* (L.) Osbeck juice, or *Salvia officinalis* L. leaves are added. In this case, she underlines that it is just for flavoring. Sometimes, she adds Marseille soap to give the prepared remedy a creamy texture or EtOH as a preservative.	Decoction	Oral	Stomach ache	
			Decoction	Compress	Anti-inflammatory, disinfectant, and wound healing	The infusion/decoction hybrid is used as an external compress to treat wounds and sores.
			Decoction	External	Anti-inflammatory, emollient, soothing (in newborns and babies)	The ‘cream’ obtained mixing the decoction with Marseille soap is used to treat children’s thrush.
			Decoction	Gargles	Gingivitis, toothache, abscesses, mouth ulcers	
			Decoction	Oral	Laxative, bowel movement	
			Decoction	Oral	Cystitis and other urinary inflammations	In this specific case, cherry stalks (*Prunus avium* (L.) L.) are added to the decoction.
			Decoction	Vaginal douche	Cystitis and other urinary inflammations	It was used to treat delivery wounds (she remembers that this use was recommended by old-time midwives)
	SAN006	61 yo. Daughter of SAN005. She knows all the uses of agrimony thanks to SAN005, and she also still uses it abundantly.	Decoction	Oral	Stomach ache	See SAN005
			Decoction	Compress	Anti-inflammatory, disinfectant, and wound healing	See SAN005
			Decoction	External	Anti-inflammatory, emollient, soothing (in newborns and babies)	See SAN005
			Decoction	Gargling	Gingivitis, toothache, abscesses, mouth ulcers	See SAN005
			Decoction	Oral	Laxative, bowel movement	See SAN005
			Decoction	Oral	Cystitis and other urinary inflammations	See SAN005
			Decoction	Vaginal douche	Cystitis and other urinary inflammations	See SAN005
Leaves	FUI015	53 yo, born in Corna Imagna. Anything she knows (including the use of ‘*Erba del Vinil*’) she learned from her grandfather.	As it is	External	Anti-inflammatory, disinfectant, and wound healing	She applied it fresh on the wounds because her grandfather told her that it would work.
	COR010	85 yo.	As it is	External	Anti-inflammatory, disinfectant, and wound healing	
			Infusion	Compress	Anti-inflammatory, disinfectant, and wound healing	
	BRU001	76 yo.	Infusion	Oral	Generic anti-inflammatory	It is advisable to drink the infusion after it cooled down in the morning for 7 days.
			Infusion	Oral	Prostate	It is advisable to drink the infusion after it cooled down in the morning for 7 days.
			Infusion	Oral	Contusions	
	LOC002	71 yo. He does not remember much, but he remembers that the plant was used also to treat animals. He knows the vernacular name. He recalls its use when he was a child, and his parents and grandparents would send him in the fields to collect it.	Infusion	Compresses	Anti-inflammatory, disinfectant, and wound healing	
	COR024	65 yo.	Infusion	Compresses	Anti-inflammatory, disinfectant, and wound healing	
	LOC021	82 yo.	Infusion	Oral	Generic anti-inflammatory	It was drunk once, when ‘they were not feeling very well’.
			As it is	External	Anti-inflammatory, disinfectant, and wound healing	
	**COS001**	69 yo. Local medical doctor. He remembers that, when he was a child, everyone would know and use the plant.	As it is	External	Anti-inflammatory, disinfectant, and wound healing	The leaf was collected fresh and directly applied on the wounds. Then, it was set in place with a gauze. The leaf was changed frequently, and the procedure was repeated for at least 3–4 days. He remembers the use very well. He remembers his parents, grandparents, and other elders of the community using this remedy when he was a child.

**Table 2:** Results of the histochemical tests on the leaf glandular trichomes of *A. eupatoria* subsp. eupatoria.

Stainings	Target Compounds	Rounded Capitate	Cylindrical Capitate
Fluoral Yellow-088	Total lipids	**+**	+
Nile Red	Neutral lipids	**+**	+
Nadi reagent	Terpenoids	**++**	++
Ruthenium Red	Acid polysaccharides	**−**	−
Alcian Blue	Muco-polysaccharides	**−**	−
Ferric Trichloride	Polyphenols	**−**	++

Symbols: (−) negative response; (+) positive response; (++) intensely positive response.

**Table 4:** Composition of phenolic molecules in the agrimony extracts. The table reports the content of molecules, expressed as µg g^−1^ DW, in different extracts obtained from different plant material from *A. eupatoria*. The results are expressed as the mean ± SE (n = 3). AgrEpi35: aqueous epicuticular extract (35 °C) from whole agrimony leaves. AgrInfL/AgrInfP: infusion obtained from grounded agrimony leaves/plants. AgrDecL/AgrDecP: decoction obtained from grounded agrimony leaves/plants. Trace: amounts under the quantification threshold (1 ng/mL). -: not detected. Statistics: comparison among infusions and decotions. *p*(E): *p* value of the effect of the extraction method; *p*(M): *p* value of the effect of the plant material; *p*(I): *p* value of the interaction; different letters indicate significant differences. n.s.: not significant. Sum: calculated as the sum of the amounts of the molecules belonging to each subclass. Total amount: calculated as the sum of the amounts of all the molecules.

N.	Compound	AgrEpi35 µg g^−1^ DW	AgrInfL µg g^−1^ DW	AgrDecL µg g^−1^ DW	AgrInfP µg g^−1^ DW	AgrDecP µg g^−1^ DW	Statistics		
							*p*(E)	*p*(M)	*p*(I)
*Simple phenols*									
1	Gallic acid	-	-	370.45 ± 89.99	-	349.23 ± 60.83	<0.001		
2	Hydroxybenzoic acid	-	51.18 ± 12.71 (b)	100.53 ± 6.72 (a)	34.71 ± 0.98 (b)	25.24 ± 4.35 (b)			0.005
3	Vanillic acid	-	188.64 ± 53.86	222.77 ± 9.50	171.60 ± 9.60	148.41 ± 30.65	n.s.		
	Sum		239.83 ± 66.33	693.76 ± 102.86	206.31 ± 8.70	522.89 ± 92.68	0.001		
*Phenolic acid derivatives*									
6	α-1-caffeoylglucose	-	39.79 ± 3.14	39.06 ± 6.91	12.13 ± 1.55	9.54 ± 2.41		<0.001	
7	β-6-caffeoylglucose	-	20.46 ± 3.08	21.82 ± 2.05	5.79 ± 0.37	-		<0.001	
11	α-6-caffeoylglucose	-	21.11 ± 3.55	23.88 ± 1.77	-	-		<0.001	
5	Neochlorogenic acid	-	735.02 ± 228.50	449.79 ± 31.76	130.1 ± 29.95	75.4 ± 16.89		0.003	
14	4-O-caffeoylquinic acid	-	178.04 ± 42.55 (b)	562.74 ± 51.50 (a)	48.46 ± 8.11 (b)	74.78 ± 20.84 (b)			<0.001
13	Coumaroyl acid hexoside	-	27.32 ± 1.34 (a)	- (b)	- (b)	- (b)			<0.001
8	3-O-*p*-Coumaroylquinic acid	-	200.20 ± 53.93	120.16 ± 13.11	40.65 ± 5.45	19.17 ± 3.68		0.002	
19	5-O-*p*-Coumaroylquinic acid	-	128.2 ± 24.00 (b)	435.52 ± 51.46 (a)	32.89 ± 3.12 (b)	48.67 ± 13.18 (b)			0.001
	Sum		1350.18 ± 356.51	1653.02 ± 155.17	270.04 ± 35.86	227.59 ± 56.43		<0.001	
*Flavonol derivatives*									
25	Hyperoside	-	206.94 ± 29.15 (b)	743.24 ± 49.46 (a)	594.75 ± 105.9 (a)	706.1 ± 71.95 (a)			0.016
26	Quercetin 3-glucoside	-	163.88 ± 30.78 (b)	546.73 ± 41.34 (a)	129.96 ± 27.13 (b)	173.93 ± 38.17 (b)			0.001
22	Quercetin 3-O-rhamnoside-7-O-glucoside	-	111.54 ± 19.93	188.38 ± 8.07	169.54 ± 23.76	186.4 ± 21.01	0.040		
24	Rutin	-	111.28 ± 2.87 (b)	235.79 ± 12.08 (a)	112.3 ± 10.82 (b)	119.76 ± 14.01 (b)			<0.001
21	Quercetin O-galloyl-hexoside	-	70.57 ± 10.57	104.07 ± 12.27	46.44 ± 10.62	46.98 ± 6.1		0.004	
29	Kaempferol O-glucoside	TRACE	- (c)	- (c)	28.83 ± 4.29 (b)	51.47 ± 1.39 (a)			0.001
28	Kaempferol 3-O-rutinoside	-	178.97 ± 18.80	248.97 ± 2.97	129.30 ± 7.96	156.65 ± 20.44	0.010	0.001	
31	*trans*-tiliroside	-	TRACE (c)	1735.30 ± 147.33 (a)	TRACE (c)	1115.64 ± 183.71 (b)			0.030
32	*cis*-tiliroside	-	- (b)	148.55 ± 33.02 (a)	- (b)	- (b)			0.002
33	Acetyl-tiliroside (1)	-	- (b)	402.52 ± 71.87 (a)	- (b)	62.96 ± 20.13 (b)			0.002
34	Acetyl-tiliroside (2)	-	- (c)	766.52 ± 116.28 (a)	- (c)	351.98 ± 57.86 (b)			0.013
	Sum		843.22 ± 108.45 (c)	5120.11 ± 387.55 (a)	1211.14 ± 184.21 (c)	2971.93 ± 420.16 (b)			0.003
*Flavone derivatives*									
23	Vitexin	TRACE	491.95 ± 96.51 (b)	1005.45 ± 77.12 (a)	69.26 ± 9.06 (c)	157.8 ± 35.27 (c)			0.011
30	Apigenin 7-O-glucoside	-	843.88 ± 137.83 (b)	1725.54 ± 172.4 (a)	250.43 ± 30.45 (c)	269.03 ± 25.32 (c)			0.005
27	Luteolin 7-O-glucoside	13.99 ± 3.43	7244.69 ± 497.5	9904.41 ± 624.6	3276.21 ± 534.22	4414.32 ± 314.34	0.006	<0.001	
	Sum		8580.54 ± 715.85	12,635.41 ± 854.84	3595.92 ± 572.50	4841.16 ± 366.43	0.004	<0.001	
*Flavan-3-ol derivatives*									
9	Procyanidin B1	-	313.56 ± 91.03	609.86 ± 74.42	442.58 ± 54.01	590.83 ± 15.94	0.009		
10	Procyanidin B3	-	-	-	625.99 ± 70.98	685.44 ± 57.66		<0.001	
12	Catechin	2.93 ± 1.62	846.67 ± 175.19	1216.99 ± 101.00	1102.45 ± 107.62	1604.06 ± 85.46	0.007	0.030	
15	Procyanidin C2	-	28.6 ± 3.44 (c)	73.54 ± 4.37 (a)	56.95 ± 5.31 (b)	77.22 ± 2.54 (a)			0.016
16	Procyanidin tetramer	-	1.45 ± 0.50	10.5 ± 0.62	-	9.89 ± 0.22	<0.001	0.039	
17	Procyanidin B2	-	60.88 ± 10.43 (b)	137.87 ± 8.36 (a)	94.59 ± 18.62 (ab)	- (c)			<0.001
18	Epicatechin	2.17 ± 0.89	489.91 ± 85.77 (ab)	644.89 ± 35.52 (a)	552.73 ± 77.79 (ab)	255.68 ± 45.22 (b)			0.008
20	Procyanidin C1	-	15.45 ± 3.89 (c)	43.12 ± 0.73 (a)	25.42 ± 2.61 (b)	- (d)			<0.001
	Sum		1756.53 ± 365.77	2736.78 ± 185.63	2900.75 ± 332.67	3223.15 ± 134.79	0.044	0.017	
*Ellagitannins*									
4	Pedunculagin	-	- (c)	- (c)	4.06 ± 0.96 (c)	17.24 ± 2.28 (a)			<0.001
	Total amount		12,770.3 ± 1516.3 (b)	22,839.1 ± 1468.8 (a)	8188.2 ± 1099.0 (b)	11,804.0 ± 957.6 (b)			0.036

**Table 5:** Total profile of volatile organic compounds (VOCs) spontaneously emitted by fresh leaves of *A. eupatoria* subsp. *eupatoria* collected in Valle Imagna (BG, Italy).

	RT	RI	Subclass	Compound	RA%
**1**	9.36	931.32	MH	α-Thujene	0.12
**2**	9.56	938.03	MH	(+)-α-Pinene	81.39
**3**	9.60	939.48	MH	α-Fenchene	0.04
**4**	9.98	952.45	MH	Camphene	0.12
**5**	10.18	958.94	MH	Dehydrosabinene	0.21
**6**	10.79	979.70	MH	β-Thujene	3.92
**7**	11.25	995.09	MH	*p*-Mentha-1(7),8-diene	0.39
**8**	11.44	1003.05	MH	α-Phellandrene	0.65
**9**	11.69	1019.59	MH	(+)-4-Carene	0.09
**10**	11.85	1029.82	OTHER	1-methyl-2-propan-2-ylbenzene	1.68
**11**	11.91	1033.86	MH	(−)-Limonene	5.36
**12**	12.05	1043.40	OTHER	*o*-Cresol	0.16
**13**	12.38	1064.67	OTHER	γ-Terpinene	0.15
**14**	12.44	1068.61	OTHER	Artemesia ketone	1.39
**15**	12.79	1091.75	MH	α-Terpinolene	0.32
**16**	12.84	1094.45	OTHER	Dehydro-*p*-cymene	0.29
**17**	12.99	1106.30	OTHER	Nonanal	0.02
**18**	13.27	1132.56	OM	Campholenic aldehyde	0.05
**19**	13.38	1142.80	OTHER	Cosmene	0.03
**20**	13.48	1152.14	OM	*trans*-2-Caren-4-ol	0.04
**21**	13.65	1168.17	OM	Isopinocamphone	0.05
**22**	13.72	1174.40	OM	Isoborneol	0.03
**23**	14.02	1203.10	OTHER	Methyl salicylate	0.09
**24**	14.15	1218.41	OM	(+)-Verbenone	0.04
**25**	14.43	1251.86	OM	Cuminaldehyde	0.03
**26**	15.29	1362.47	OTHER	Trimethylsilyl nonanoate	0.14
**27**	15.35	1370.20	SH	α-Cedrene	0.10
**28**	15.51	1392.07	SH	(7S,10R)-10-methyl-4-methylidene-7-propan-2-yltricyclo[4.4.0.01,5]decane	0.16
**29**	15.77	1429.63	SH	(−)-α-Gurjunene	0.03
**30**	15.85	1441.71	SH	1,1,7-trimethyl-4-methylidene-2,3,4a,5,6,7,7a,7b-octahydro-1aH-cyclopropa[e]azulene	0.20
**31**	15.96	1458.76	OTHER	trimethylsilyl decanoate	0.04
**32**	15.98	1461.85	SH	(6Z)-7,11-dimethyl-3-methylidenedodeca-1,6,10-triene	0.08
**33**	15.99	1463.03	SH	β-Humulene	0.01
**34**	16.08	1476.54	OTHER	β-Acoradiene	0.31
**35**	16.12	1481.75	SH	α-Curcumene	0.07
**36**	16.19	1491.46	SH	α-Bisabolene	0.15
**37**	16.20	1494.07	SH	(+)-Cuparene	1.06
**38**	16.35	1515.87	SH	(3S,4aS,5R)-4a,5-dimethyl-3-prop-1-en-2-yl-2,3,4,5,6,7-hexahydro-1H-naphthalene	0.12
**39**	16.50	1538.95	SH	Selina-3,7(11)-diene	0.11
**40**	16.52	1541.60	SH	*cis*-Calamenene	0.26
**41**	16.94	1605.55	OS	Spathulenol	0.05
**42**	17.35	1661.77	OTHER	*cis*-7-Tetradecen-1-ol	0.05
**43**	17.47	1679.19	OS	Longiverbenone	0.32
**44**	18.30	1775.23	OS	(+)-Nootkatone	0.08
				Total	100.00
			MH	Monoterpene hydrocarbon	92.60
			OM	Oxygenated monoterpene	0.24
			SH	Sesquiterpene hydrocarbon	2.36
			OS	Oxygenated sesquiterpene	0.45
			OTHER	Other	4.35

RT = retention time; RI = Kovats Retention Index; RA% = relative abundance; OTHER = benzoic acids and derivatives, branched unsaturated hydrocarbons, carbonyl compounds, cresols, cumenes, fatty alcohols, olefins, organosilicon compounds, and phenylpropenes.

**Table 6:** Macroscopic diagnostic features for the determination of *A. eupatoria* subsp. *eupatoria*. Characteristics with highest taxonomic value are in bold [2,3].

***Hypanthium*/crown length ratio > 1**
**Lowest bristles erect to erect-patent; angled *hypanthium*/lowest bristles < 45°**
Length of *hypanthium* + crown 7.5–12.5 (–13.5) mm
Stem thin [2–4 (–5)], glabrous or nearly so with glandular hairs concealed by the non-glandular pubescence
Basal rosette present (occasionally absent)

## Data Availability

This paper contains the complete data concerning our investigation on *Agrimonia eupatoria* L. collected in Valle Imagna.

## Supplementary Materials

The following supporting information can be downloaded at: https://www.mdpi.com/article/10.3390/plants14030340/s1, Figure S1. Chromatographic profiles of the extract AgrInfL. The figure shows the automatically integrated Extracted Ion Chromatograms peaks (grey filled) of the identified molecule. Peak numbering refers to the main text (Table 3 and Table 4). Figure S2. Chromatographic profiles of the extract AgrDecL. The figure shows the automatically integrated Extracted Ion Chromatograms peaks (grey filled) of the identified molecule. Peak numbering refers to the main text (Table 3 and Table 4). Figure S3. Chromatographic profiles of the extract AgrInfP. The figure shows the automatically integrated Extracted Ion Chromatograms peaks (grey filled) of the identified molecule. Peak numbering refers to the main text (Table 3 and Table 4). Figure S4. Chromatographic profiles of the extract AgrDecP. The figure shows the automatically integrated Extracted Ion Chromatograms peaks (grey filled) of the identified molecule. Peak numbering refers to the main text (Table 3 and Table 4). Figure S5. Chromatographic profiles of the extract AgrInfL. The figure shows the automatically integrated Extracted Ion Chromatograms peaks (grey filled) of the identified molecule. Peak numbering refers to the main text (Table 3 and Table 4). Figure S6. *A. eupatoria* effect on growth in *S. warneri* and *S. aureus* strains. (a,e) AgrInfL (infusion leaves), (b,f) AgrDecL (decoction leaves), (c,g) AgrInfP (infusion aerial parts), (d,h) AgrDecP (decoction aerial parts). The control without compounds is YESCA or TSB. Results of at least 3 independent biological replicates are reported, with mean and SD displayed. *, *p*-value < 0.05; one-way ANOVA with Dunnett’s test for multiple comparisons. Figure S7. *Herbarium* specimen of *A. eupatoria* deposited at the Museum of Natural History of Milan (Part A; Code: MSNM 54011). Figure S8. *Herbarium* specimen of *A. eupatoria* deposited at the Museum of Natural History of Milan (Part B; Code: MSNM 54012).
