# Supplementary material for: From Traditional Medicine to the Laboratory: A Multidisciplinary Investigation on Agrimonia eupatoria L. Collected in Valle Imagna (BG, North of Italy)
Source: Plants (Basel). 2025 Jan 23;14(3):340. doi: 10.3390/plants14030340 (PMC11819889; doi:10.3390/plants14030340)

plants

MDPI

Article

From Traditional Medicine to the Laboratory: A Multidisciplinary Investigation on *Agrimonia eupatoria* L. Collected in Valle Imagna (BG, North of Italy)

Fabrizia Milani [1,2], Chiara Muratore [3], Sara Biella [4], Martina Bottoni [1,2,*], Elio Rossi [4], Lorenzo Colombo [1,2], Paola Sira Colombo [1,2], Piero Bruschi [5], Alessio Papini [6], Paolo Landini [4], Claudia Giuliani [1,2], Fabrizio Araniti [3], Bhakti Prinsi [3] and Gelsomina Fico [1,2]

1 Department of Pharmaceutical Sciences, Università degli Studi di Milano, Via Luigi Mangiagalli 25, 20133 Milan, Italy; fabrizia.milani@unimi.it (F.M.); lorecolo.93@gmail.com (L.C.); pasico19@virgilio.it (P.S.C.); claudia.giuliani@unimi.it (C.G.); gelsomina.fico@unimi.it (G.F.)
2 "G.E. Ghirardi" Botanical Garden, Department of Pharmaceutical Sciences, Università degli Studi di Milano, Via Religione 25, 25088 Toscolano Maderno, Italy
3 Department of Agricultural and Environmental Sciences—Production, Landscape, Agroenergy, Università degli Studi di Milano, Via Celoria 2, 20133 Milan, Italy; chiara.muratore@unimi.it (C.M.); fabrizio.araniti@unimi.it (F.A.); bhakti.prinsi@unimi.it (B.P.)
4 Department of Biosciences, Università degli Studi di Milano, Via Celoria 26, 20133 Milan, Italy; sara.biella@unimi.it (S.B.); elio.rossi@unimi.it (E.R.); paolo.landini@unimi.it (P.L.)
5 Department of Agricultural, Environmental, Food and Forestry Science and Technology, Università degli Studi di Milano, Piazzale delle Cascine 18, 50144 Florence, Italy; piero.bruschi@unifi.it
6 Department of Biology, Università degli Studi di Firenze, Via La Pira 4, 50121 Florence, Italy; alessio.papini@unifi.it

* Correspondence: martina.bottoni@unimi.it[1]Department of Pharmaceutical Sciences, Università degli Studi di Milano, Via Luigi Mangiagalli 25, 20133 Milan, Italy; fabrizia.milani@unimi.it; lorecolo.93@gmail.com; pasico19@virgilio.it; claudia.giuliani@unimi.it; gelsomina.fico@unimi.it

[2]"G.E. Ghirardi" Botanical Garden, Department of Pharmaceutical Sciences, Università degli Studi di Milano, Via Religione 25, 25088 Toscolano Maderno, Italy;

[3]Department of Agricultural and Environmental Sciences - Production, Landscape, Agroenergy, Università degli Studi di Milano, Via Celoria 2, 20133 Milan, Italy; chiara.muratore@unimi.it; bhakti.prinsi@unimi.it; fabrizio.araniti@unimi.it

[4]Department of Biosciences, Università degli Studi di Milano, Via Celoria 26, 20133 Milan, Italy; paolo.landini@unimi.it; elio.rossi@unimi.it; sara.biella@unimi.it

[5]Department of Agricultural, Environmental, Food and Forestry Science and Technology, Università degli Studi di Firenze, Piazzale delle Cascine 18, 50144 Florence, Italy; piero.bruschi@unifi.it

[6]Department of Biology, Università degli Studi di Firenze, Via La Pira 4, 50121, Florence, Italy; alessio.papini@unifi.it

[*]Corresponding author: martina.bottoni@unimi.it

Academic Editor: Juei-Tang Cheng

Received: 10 December 2024
Revised: 20 January 2025
Accepted: 21 January 2025
Published: 23 January 2025

Citation: Milani, F.; Muratore, C.; Biella, S.; Bottoni, M.; Rossi, E.; Colombo, L.; Colombo, P.S.; Bruschi, P.; Papini, A.; Landini, P.; et al. From Traditional Medicine to the Laboratory: A Multidisciplinary Investigation on Agrimonia eupatoria L. Collected in Valle Imagna (BG, North of Italy). *Plants* **2025**, *14*, 340. https://doi.org/10.3390/plants14030340

Copyright: © 2025 by the authors. Licensee MDPI, Basel, Switzerland. This article is an open access article distributed under the terms and conditions of the Creative Commons Attribution (CC BY) license (https://creativecommons.org/licenses/by/4.0/).

plants

MDPI

Figure S1. Chromatographic profiles of the extract AgrInfL. The figure shows the automatically integrated Extracted Ion Chromatograms peaks (grey filled) of the identified molecule. Peak numbering refers to the main text (Tables 3 and 4).

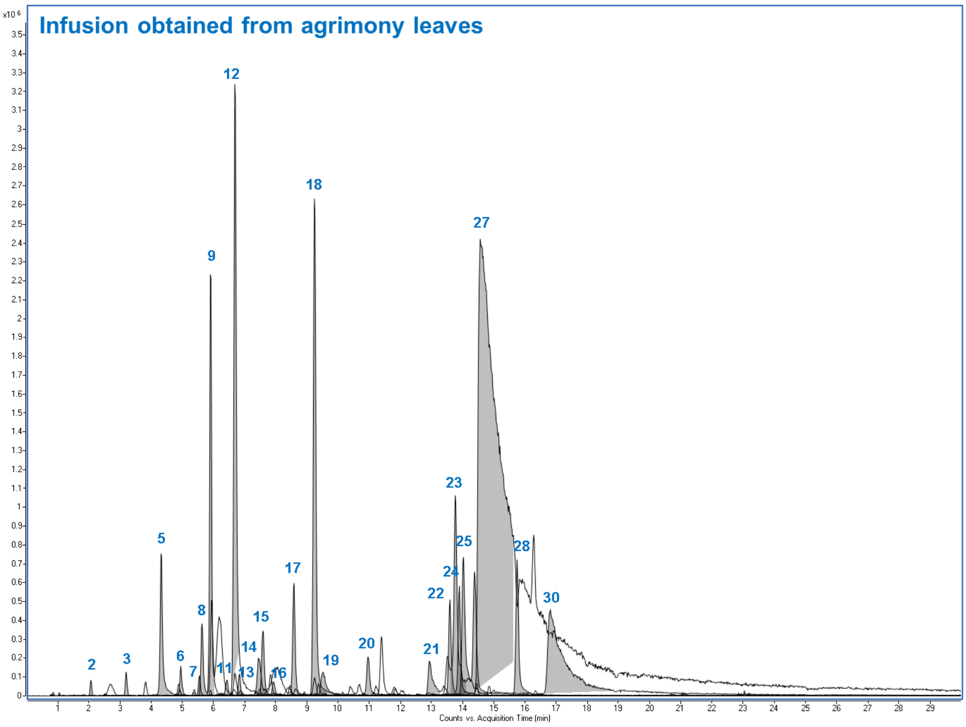

Figure S2. Chromatographic profiles of the extract AgrDecL. The figure shows the automatically integrated Extracted Ion Chromatograms peaks (grey filled) of the identified molecule. Peak numbering refers to the main text (Tables 3 and 4).

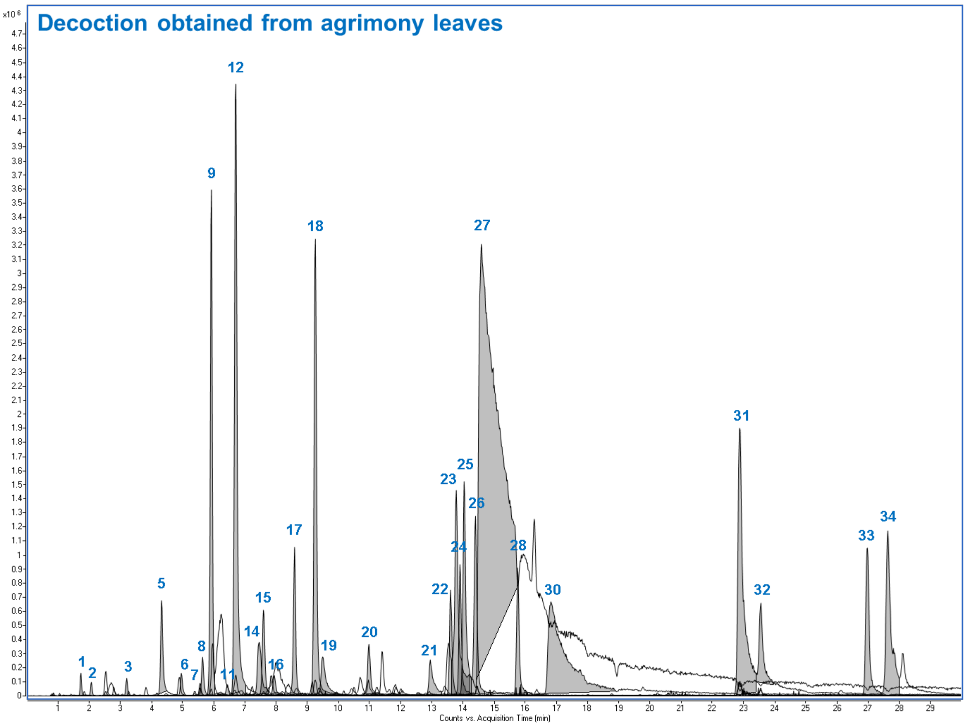

Figure S3. Chromatographic profiles of the extract AgrInfP. The figure shows the automatically integrated Extracted Ion Chromatograms peaks (grey filled) of the identified molecule. Peak numbering refers to the main text (Tables 3 and 4).

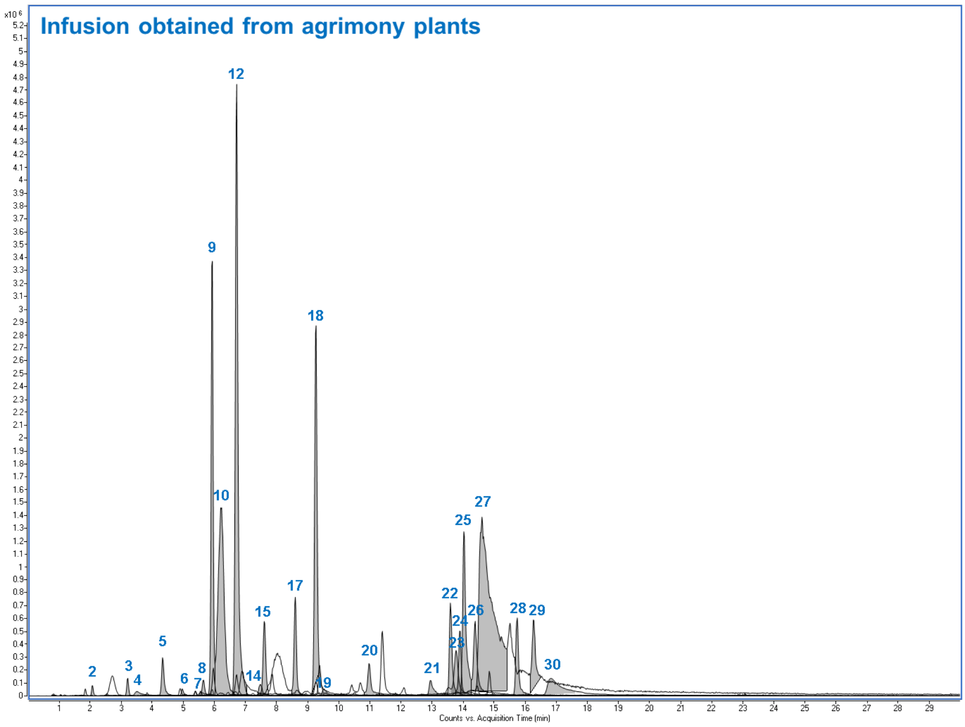

Figure S4. Chromatographic profiles of the extract AgrDecP. The figure shows the automatically integrated Extracted Ion Chromatograms peaks (grey filled) of the identified molecule. Peak numbering refers to the main text (Tables 3 and 4).

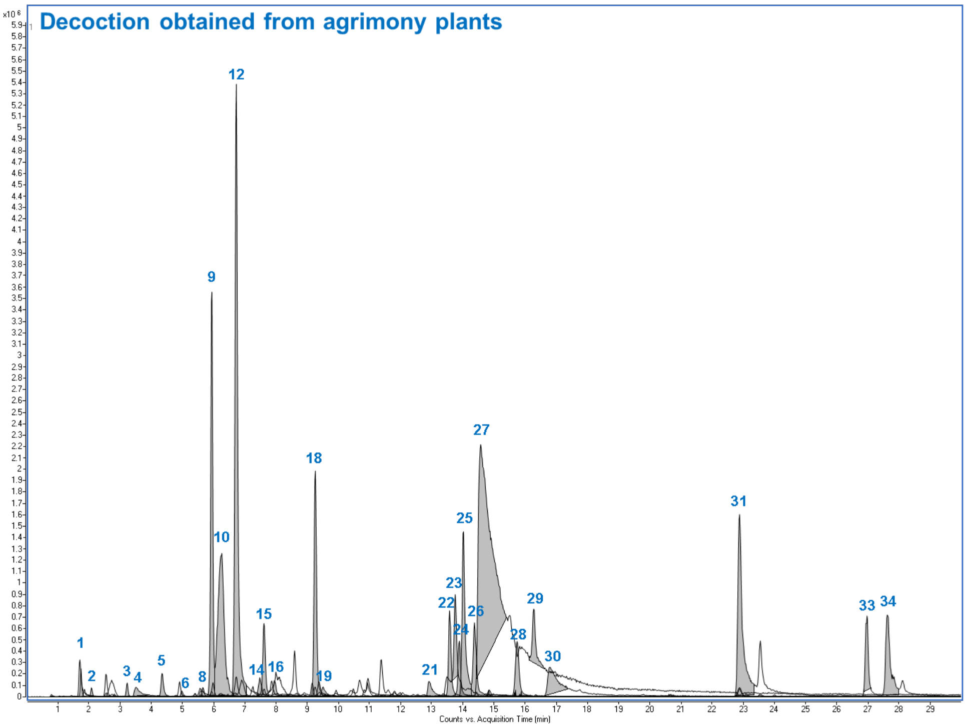

Figure S5. Chromatographic profiles of the extract AgrInfL. The figure shows the automatically integrated Extracted Ion Chromatograms peaks (grey filled) of the identified molecule. Peak numbering refers to the main text (Tables 3 and 4).

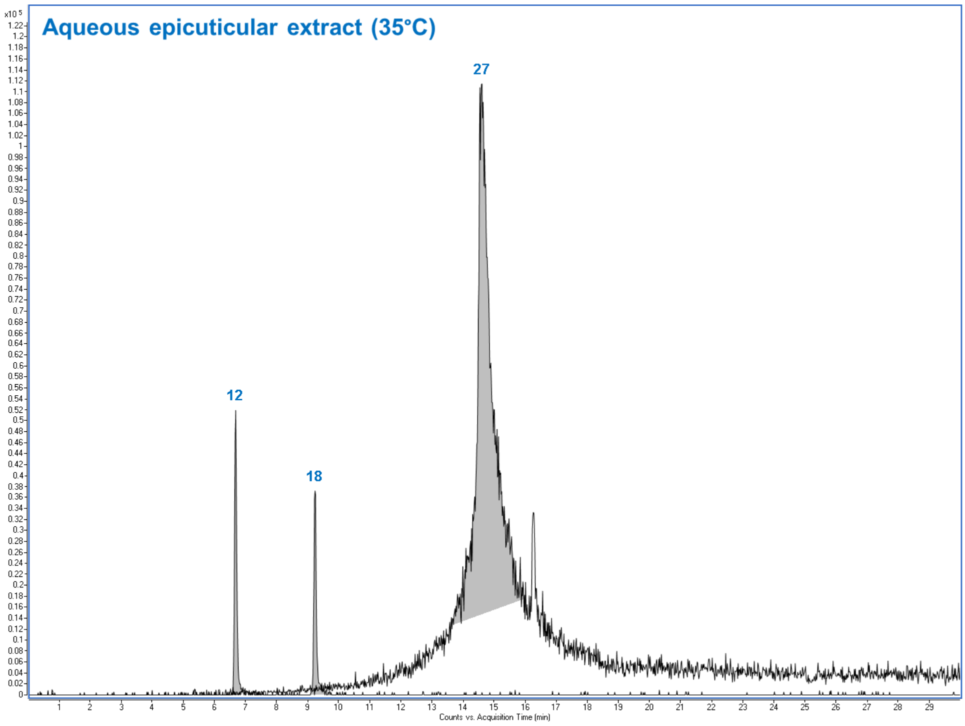

Figure S6. *A. eupatoria* effect on growth in *S. warneri* and *S. aureus* strains. (**a, e**) AgrInfL (infusion leaves), (**b, f**) AgrDecL (decoction leaves), (**c, g**) AgrInfP (infusion aerial parts), (**d, h**) AgrDecP (decoction aerial parts). The control without compounds is YESCA or TSB. Results of at least 3 independent biological replicates are reported, with mean and SD displayed. *, p-value <0.05, one-way ANOVA with Dunnett's test for multiple comparisons.

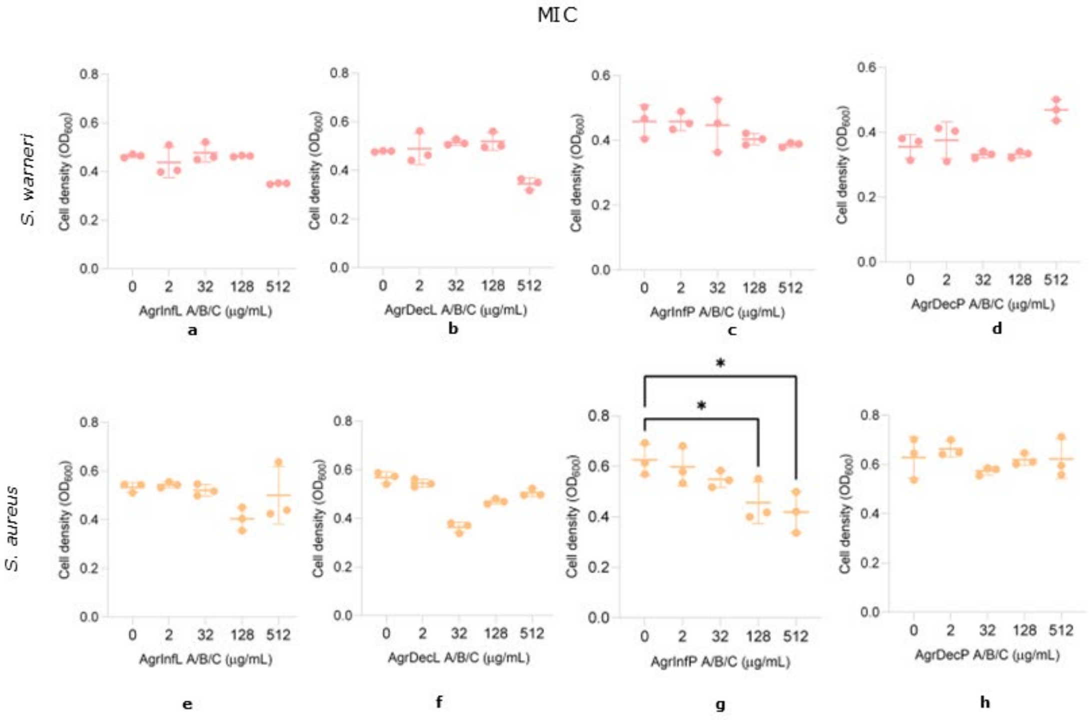

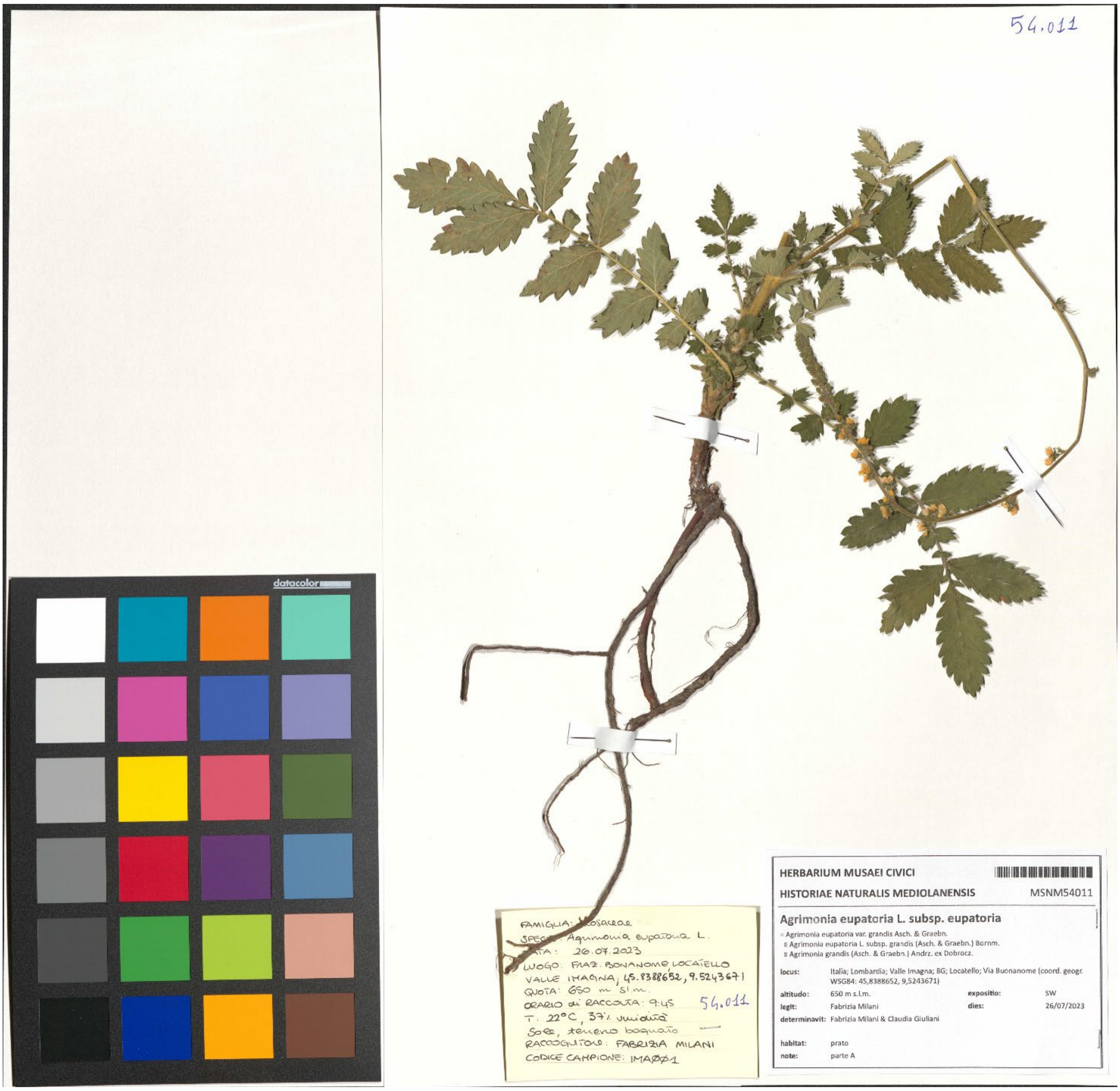

Figure S7. *Herbarium* specimen of *A. eupatoria* deposited at the Museum of Natural History of Milan (Part A; Code: MSNM 54011).

plants

MDPI

Figure S8. Herbarium specimen of *A. eupatoria* deposited at the Museum of Natural History of Milan (Part B; Code: MSNM 54012).

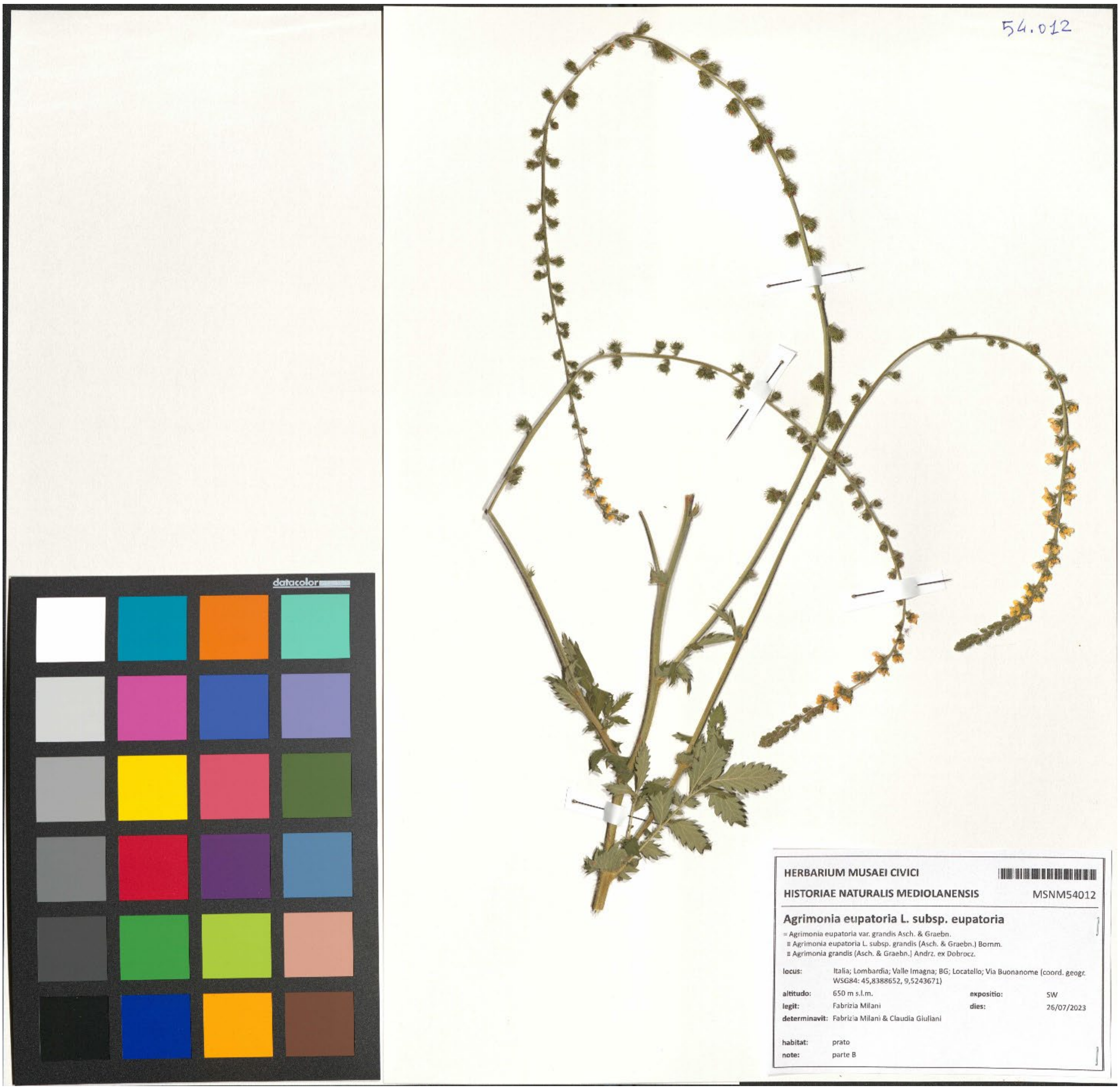

Supplement: Supplementary file 1 [file plants-14-00340-s001.zip › plants-3391658-supplementary.pdf]
